# Expanded Dataset Reveals the Emergence and Evolution of DNA Gyrase in Archaea

**DOI:** 10.1093/molbev/msac155

**Published:** 2022-07-09

**Authors:** Paul Villain, Ryan Catchpole, Patrick Forterre, Jacques Oberto, Violette da Cunha, Tamara Basta

**Affiliations:** Université Paris-Saclay, CEA, CNRS, Institute for Integrative Biology of the Cell (I2BC), 91198 Gif-sur-Yvette, France; Université Paris-Saclay, CEA, CNRS, Institute for Integrative Biology of the Cell (I2BC), 91198 Gif-sur-Yvette, France; Université Paris-Saclay, CEA, CNRS, Institute for Integrative Biology of the Cell (I2BC), 91198 Gif-sur-Yvette, France; Archaeal Virology Unit, Institut Pasteur, Paris, France; Université Paris-Saclay, CEA, CNRS, Institute for Integrative Biology of the Cell (I2BC), 91198 Gif-sur-Yvette, France; Université Paris-Saclay, CEA, CNRS, Institute for Integrative Biology of the Cell (I2BC), 91198 Gif-sur-Yvette, France; Université Paris-Saclay, CEA, CNRS, Institute for Integrative Biology of the Cell (I2BC), 91198 Gif-sur-Yvette, France

**Keywords:** Archaea, horizontal gene transfer, DNA gyrase, topoisomerase evolution, Topoisomerase VI

## Abstract

DNA gyrase is a type II topoisomerase with the unique capacity to introduce negative supercoiling in DNA. In bacteria, DNA gyrase has an essential role in the homeostatic regulation of supercoiling. While ubiquitous in bacteria, DNA gyrase was previously reported to have a patchy distribution in Archaea but its emergent function and evolutionary history in this domain of life remains elusive. In this study, we used phylogenomic approaches and an up-to date sequence dataset to establish global and archaea-specific phylogenies of DNA gyrases. The most parsimonious evolutionary scenario infers that DNA gyrase was introduced into the lineage leading to Euryarchaeal group II via a single horizontal gene transfer from a bacterial donor which we identified as an ancestor of Gracilicutes and/or Terrabacteria. The archaea-focused trees indicate that DNA gyrase spread from Euryarchaeal group II to some DPANN and Asgard lineages via rare horizontal gene transfers. The analysis of successful recent transfers suggests a requirement for syntropic or symbiotic/parasitic relationship between donor and recipient organisms. We further show that the ubiquitous archaeal Topoisomerase VI may have co-evolved with DNA gyrase to allow the division of labor in the management of topological constraints. Collectively, our study reveals the evolutionary history of DNA gyrase in Archaea and provides testable hypotheses to understand the prerequisites for successful establishment of DNA gyrase in a naive archaeon and the associated adaptations in the management of topological constraints.

## Introduction

Topoisomerases are enzymes which control DNA topology in every living cell. By their transient DNA cleaving activities, they maintain DNA supercoiling in a range compatible with DNA transactions such as transcription and DNA replication and ensure decatenation of chromosomes prior to cell division (Wang 2002; Schoeffler and Berger 2008; Forterre 2011; Pommier 2012; Bush et al. 2015; Seol and Neuman 2016; McKie et al. 2021). Topoisomerases are classified as type I or type II depending on whether they cleave one or two DNA strands (Schoeffler and Berger 2008). In principle, only one type I and one type II topoisomerase would be necessary and sufficient for resolving the accumulation of DNA supercoils and DNA entanglements occurring naturally in cells. Intriguingly, however, there are multiple subclasses of each topoisomerase type, distributed unequally among the three domains of life and divided by both amino-acid sequence and reaction mechanism. This puzzling distribution and variety of topoisomerase families cannot be easily reconciled with the classical evolution of organisms resolving into the three domains of life. The identity of the most ancient original topoisomerases and the evolutionary pathway leading to the present-day diversity and distribution remains one of the major evolutionary enigmas with implications for understanding the evolution of DNA-based genomes (Forterre et al. 2007; Forterre and Gadelle 2009).

All topoisomerases have the capacity to relax supercoiled DNA but only two are also able to actively introduce supercoils, converting relaxed DNA into supercoiled DNA. Reverse gyrase can supercoil DNA in a positive manner—a function seemingly essential for life at high temperature as this enzyme has been systematically found in all hyperthermophiles and in many thermophiles but never in mesophiles (Forterre 2002; Catchpole and Forterre 2019). DNA gyrase, an antagonist of reverse gyrase, catalyzes the ATP-dependent introduction of negative supercoils in constrained DNA molecules (Gellert et al. 1976). Due to the inherent nature of DNA unwinding, transcribing RNA polymerase complexes and DNA replication complexes accumulate positive supercoils ahead of their direction of travel. If left unchecked, this torsion will result in stalling of the transcription/replication complex. According to the twin supercoiled-domain model, DNA gyrase removes these positive supercoils thus allowing these essential processes to proceed (Liu and Wang 1987; Drlica 1992; Lal et al. 2016; Stracy et al. 2018; Sutormin et al. 2018). In tandem with topoisomerase I (Topo I), DNA gyrase regulates the global supercoiling level in bacterial cells, with even small deviations from optimal DNA topology being lethal for bacteria (Pruss et al. 1982).

DNA gyrase activity (and thus the supercoiling state of DNA) is directly linked with the global state of cellular metabolism *via* the intracellular ATP/ADP ratio. In stressful conditions such as nutrient depletion, cellular ATP levels decrease and render the ATP-dependent DNA gyrase-less active. As a result, the global level of DNA supercoiling becomes less negative. In response to this topological change, the activity of numerous promoters and transcriptional regulators (controlling up to 48% of all genes) is simultaneously altered, allowing rapid adaptation to such unfavorable conditions (Westerhoff et al. 1988; Hsieh et al. 1991; Bush et al. 2015; Dorman and Dorman 2016; Martis et al. 2019). The capacity of DNA gyrase to quickly translate environmental stimuli into an appropriate global transcriptional response confers a key evolutionary advantage to Bacteria, providing the capacity to adapt rapidly to a changing environment (Tse-Dinh 2003; Dorman 2006; Forterre and Gadelle 2009).

**Fig. 1.**
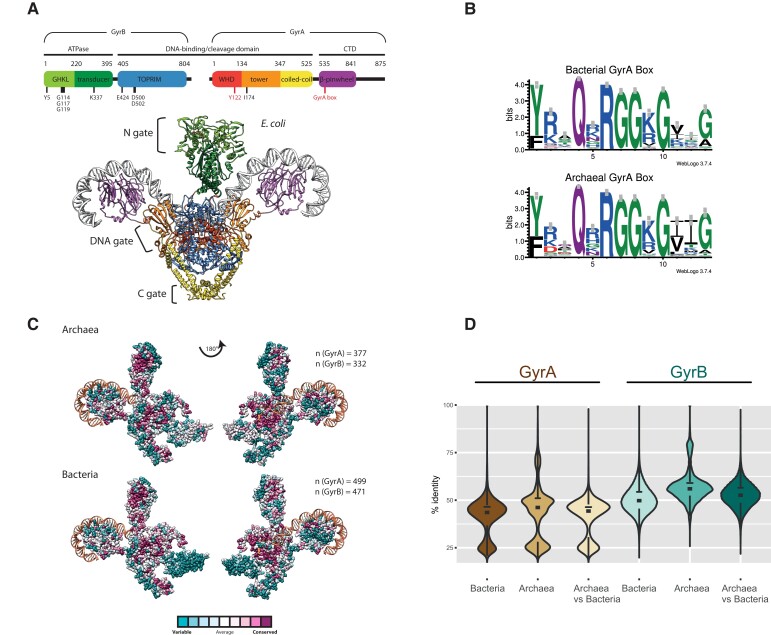
Sequence conservation of archaeal and bacterial DNA gyrases. (*A*) Structure of *Escherichia coli* DNA gyrase. A schematic representation of functional domains and catalytically important residues in GyrA and GyrB is shown. The GyrA box motif required for negative supercoiling activity and the catalytic tyrosine Tyr122 are highlighted in red. The three-dimensional structure of the A_2_B_2_ heterotetramer (PDB Nr. 6RKW) is shown bound to a 130 bp DNA duplex (same color code as the above scheme). The DNA duplex is depicted in grey. Gate regions are indicated to the left. (*B*) Comparison of the GyrA box motif in DNA gyrase sequences. The motif was generated using an alignment of 499 bacterial and 377 archaeal representative sequences. The letter size is proportional to the frequency of occurrence of each letter in the alignment. WebLogo v. 3.7.4. was used to generate the sequence logo (Crooks et al. 2004). (*C*) The conservation scores of DNA gyrase amino acids were computed by ConSurf server using the empirical Bayesian method from the multiple sequence alignment. The number of aligned sequences is indicated. The ConSurf conservation score was projected onto the structure of *E. coli* DNA gyrase (PDB 6RKW). For clarity, only one A and one B subunits are shown.(*D*) Sequence conservation analysis using pairwise BLAST. Sequence identity was determined using all against all BLASTp searches. The statistical analysis and graphical representation were generated using R packages (see Materials and Methods). The analysis shows that the mean level and distribution of sequence conservation within each domain or between the two domains is similar.

Bacterial DNA gyrase A and B subunits assemble into an A_2_B_2_ heterotetramer of approximately 370 kDa forming three major subunit interfaces, or gates, called N-gate, DNA-gate, and C-gate (Fig. 1*A*). In order to perform its function, DNA gyrase undergoes a series of conformational changes that consist of concerted gate openings, DNA cleavage, and DNA strand passage events (Schoeffler and Berger 2008; Soczek et al. 2018; Vanden Broeck et al. 2019). The DNA-gate houses the catalytic tyrosine and cooperates with the TOPRIM fold to cleave DNA. The unique C-terminal DNA-binding domain (CTD) carries the conserved GyrA—box motif, Q(R/K)RGG(R/K)G, which has been identified as the defining feature of DNA gyrases. This motif is essential for chiral wrapping of DNA around gyrase, enabling these enzymes to introduce negative supercoils in relaxed or positively supercoiled DNA.

In contrast to bacterial orthologs that have been extensively studied *in vitro*, only one archaeal DNA gyrase, that of *Thermoplasma acidophilum,* has been biochemically characterized. This enzyme exhibited *in vitro* activities similar to that of bacterial homologs that is, ATP-dependent supercoiling, decatenation activities, and ATP-independent relaxation activity (Yamashiro and Yamagishi 2005). Early studies showed that DNA gyrase exhibits negative supercoiling activity *in vivo* and that this activity is essential in methanogens, halophiles, and thermoacidophiles due to their sensitivity to the gyrase-inhibiting antibiotics novobiocin and ciprofloxacin (Sioud et al. 1988a; 1988b). While indirect, some evidence points to the involvement of gyrase-derived negative DNA supercoiling in the control of gene expression in extreme halophiles: the plasmid-encoded *gyrB* gene and the chromosomally-encoded *bop* gene (encoding bacteriorodopsin) were strongly induced (up to 20-fold) in novobiocin-treated cultures of *Haloferax* (Holmes and Dyall-Smith 1991; Yang et al. 1996). Similarly, novobiocin-treatment of *Halobacterium* species stimulated expression of DNA gyrase, topoisomerase I (Topo I), and topoisomerase VI (Topo VI) indicating the involvement of DNA gyrase and supercoiling in regulating gene expression in this organism, and intriguingly, in regulating genes responsible for resolving that supercoiling (Tarasov et al. 2011). The latter enzyme, Topo VI, is nearly ubiquitous in Archaea (missing only in Thermoplasmatales where it appears to have been replaced by DNA gyrase) and is predicted to have essential functions in chromosome decatenation and in relaxing positive supercoils accumulated during transcription and DNA replication (Forterre and Gadelle 2009; Raymann et al. 2014). As such, the activity of Topo VI overlaps with that of DNA gyrase (which canonically relaxes positive supercoils in Bacteria) suggesting some redundancy, or functional separation in gyrase-encoding Archaea yet to be investigated.

The initial phylogenomic analyzes reported that DNA gyrase was present only in Euryarchaeal group II (Forterre et al. 2007; Raymann et al. 2014) (corresponding to Gaiarchaea, *sensu*Aouad et al. 2022 , and to the clade grouping Thermoplasmatota and Halobacteriota, *sensu*Rinke et al., 2021) (Forterre et al. 2014; Rinke et al. 2021; Aouad et al. 2022). Raymann and colleagues identified DNA gyrase genes in 51 genomes of Euryarchaeal group II out of the 142 archaeal genomes available at that time. The resulting tree was not resolved for deep bacterial nodes and the archaeal sequences formed an unstable monophyletic clade dependent upon the bacterial taxonomic sampling used. With the expansion of taxonomic sampling in the recent years, DNA gyrase was also identified in several new DPANN lineages and in the Asgard archaea, but these sequences were not analyzed in the framework of a phylogenetic tree (Adam et al. 2017).

Despite the progress achieved by these studies the question of the evolutionary history of DNA gyrase in Archaea, that is the timing and the number of inter- or intra-domain horizontal transfers of DNA gyrase genes remains unanswered. Moreover, the nature of the gene-specific or genome-wide adaptations associated with DNA gyrase acquisition in recipient Archaea has not been investigated. Answering these questions is important for understanding the prerequisites for the emergence of DNA gyrase in Archaea and for testing hypotheses about the DNA topology-driven regulation of gene expression in Archaea, which is still poorly understood. To address these questions, we sampled DNA gyrase sequences from all available archaeal and bacterial genomes and performed phylogenetic, comparative sequence, and structural analyzes.

We find that DNA gyrase is present in all Euryarchaeal group II lineages, and in several new lineages of DPANN and Asgard archaea thus expanding the DNA gyrase dataset within these recently discovered Archaea. DNA gyrase is mainly absent from group I Euryarchaeota, with the exception of a sporadic presence in Theionarchaea and *Methanobrevibacter*. The global and archaeal-specific DNA gyrase tree topologies suggest an ancient transfer of DNA gyrase genes from Bacteria to the base of group II Euryarchaea, followed by secondary transfers to some DPANN and Asgard lineages. We also detected a few more recent transfers between Archaeal and Bacterial lineages (explaining the presence of DNA gyrase in some *Methanobrevibacter* archaea). Notably, we found that the global DNA gyrase tree exhibits a tripartite topology whereby Bacteria form two clades corresponding to Terrabacteria and Gracilicutes while Archaea are monophyletic. This topology suggests that the separation of Euryarchaeal group II from other Archaea predated the diversification of Terrabacteria and/or Gracilicutes. We also found some evidence, using comparative analysis of Topo VI sequences that the endogenous “topological kit” of DNA gyrase-encoding archaea may have adapted to allow successful integration of the gyrase activity into the existing system for control of DNA topology.

## Results

### Collection and Analysis of DNA Gyrase Sequences

We took advantage of the phenomenal amount of recently deposited sequence data to establish a comprehensive comparative sequence analysis of DNA gyrase in Archaea. From this, we generated a curated set of GyrA and GyrB sequences from across the entire phylogenetic diversity of Archaea and Bacteria (see Materials and Methods).

We first wished to determine whether archaeal DNA gyrase proteins have retained function similar to bacterial gyrase, or whether they may have been exapted by Archaea for other purposes. Alignment of archaeal GyrA sequences revealed the presence of all catalytically important residues in addition to the classical GyrA Box motif (fig. 1*A and B*), suggesting no loss of function, or divergence in function. Pairwise sequence identity analysis suggested that archaeal gyrases evolved at a similar rate to their bacterial counterparts (fig. 1*D*). Conservation score distribution across the functional domains of bacterial and archaeal orthologs showed that slowly evolving, functionally important positions were enriched in the ATPase and DNA-binding/cleavage domains while the C-terminal domain was less conserved, with the clear exception of the GyrA box motif (fig. 1*C*).

Together, these data indicate a similar tempo of evolution for bacterial and archaeal DNA gyrases and further suggest that the canonical negative supercoiling activity of bacterial DNA gyrases is conserved throughout the archaeal taxonomic sampling.

### Gyrase Distribution in the Archaeal Domain

**Fig. 2.**
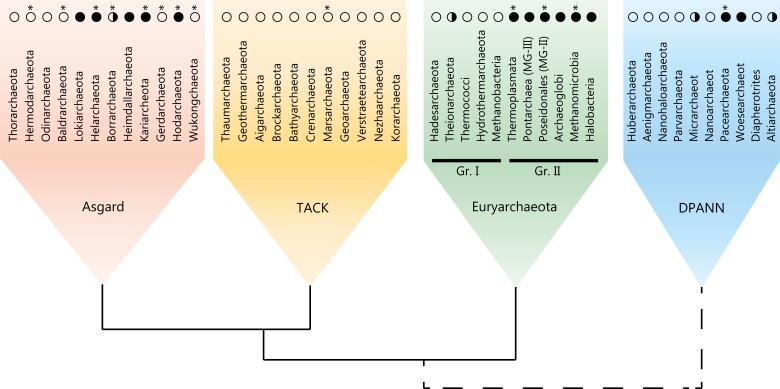
Distribution of DNA gyrase across the archaeal domain. The complete (>90%) and partial presence of *gyrA* and *gyrB* genes in a taxon are indicated with full black circle and half circle, respectively. The empty circle indicates the absence of DNA gyrase genes. It should be noted, however, that absence of genes in uncultured taxa may be due to genome incompleteness. Asterisks refer to lineages that were newly discovered or expanded (at class taxonomic level) since the last survey. The phylogenetic relationship between the four major groups is shown schematically, the uncertain position of DPANN in archaeal phylogeny is indicated with a dotted line.

Searches in public databases confirmed the systematic presence of DNA gyrase in group II Euryarchaeota and the systematic absence of this enzyme in members of the TACK superphylum (fig. 2). DNA gyrase is also almost totally absent in group I Euryarchaeota, with the exception of a sporadic presence in Theionarchaea and *Methanobrevibacter*. We detected DNA gyrase in several lineages of the DPANN superphylum: in the majority of Micrarchaeota, Woesearchaeota, Pacearchaeota, and in the recently described (and still nameless) UBA583 lineage. DNA gyrase is only sporadically present in Altiarchaeota and is completely missing in the remaining six DPANN phyla. The diversity of Asgard archaea was recently substantially expanded by the proposal of six additional phyla (Liu et al. 2021). Using BLAST searches, we detected DNA gyrase genes in Heimdallarchaeota, Kariarchaeota, and Hodarchaeota, all members of a monophyletic clade also including Gerdarchaeota and Wukongarchaeota. Gyrase is also present in distantly related Lokiarchaeota and Helarchaeota but seems to be absent in a sister monophyletic clade composed of Thorarchaeota, Hermodarchaeota, Odinarchaeota and Baldrarchaeota.

### Archaeal and Bacterial DNA Gyrases Segregate into Two Monophyletic Clades

The distribution described above could be a result of vertical inheritance of DNA gyrase genes from the Last Archaeal Common Ancestor (LACA) with multiple independent losses of DNA gyrase, or a consequence of more recent inter- and/or intra-domain lateral gene transfers to gyrase-less archaeal ancestors. In order to investigate the evolutionary history of DNA gyrase we first built a phylogenetic tree using the alignment of concatenated archaeal and bacterial GyrAB sequences. In this tree, Archaea and Bacteria were paraphyletic, with most bacterial sequences being separated into three clades while most archaeal sequences were split into two clades (see supplementary fig. S1*A*, Supplementary Material online). Few stand-alone archaeal sequences branched within bacterial phyla and *vice versa*, testifying for a very limited amount of recent HGT (horizontal gene transfer) between the two domains.

**Fig. 3.**
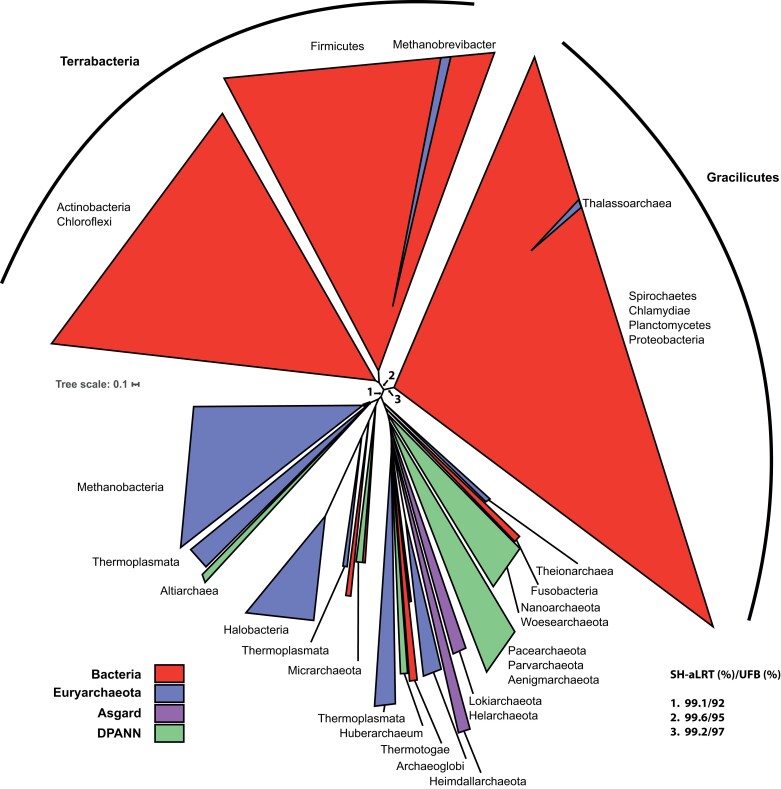
Phylogeny of archaeal and bacterial DNA gyrases. Maximum likelihood phylogenetic tree generated using the complete dataset of concatenated GyrA and GyrB sequences containing 502 bacterial and 297 archaeal sequences. The tree was inferred from an alignment trimmed with Noisy. The legend indicates the correspondence between the colors and taxonomic affiliations of the branches. SH-aLRT support (%)/ultrafast bootstrap support (%) for deep branches (1, 2, and 3) is indicated. Detailed tree upon which the schematic tree was drawn is shown in supplementary fig. S1*B*, Supplementary Material online.

Notably, the ultrafast bootstrap and SH-aLRT values (36%/36%) did not support the existence of the central branch that separated the two archaeal clades, suggesting that most archaeal sequences may fall within a single monophyletic clade. This was further suggested by the tree containing only GyrA sequences (see supplementary fig. S2, Supplementary Material online) in which these archaeal sequences formed a monophyletic clade with good branch support (97%/72%). To test this hypothesis, we modified the concatenated DNA gyrase phylogeny such that both Archaea and Bacteria would form monophyletic groups, and we used various tests of phylogenetic tree selection to estimate the likelihood of this tree. These statistical analyzes ask whether the tree consistent with the monophyly of archaea has a significantly worse likelihood score than the calculated maximum likelihood tree. The tests indicated that the monophyletic tree topology cannot be rejected (see supplementary fig. S3, Supplementary Material online). Moreover, the tree inferred using less stringent trimming algorithm showed tripartite topology with archaeal and bacterial sequences forming two monophyletic clades (fig. 3, see supplementary fig. S1*B*, Supplementary Material online). Notably, the deep branches supporting the monophyly of the three clades were now robustly supported by both UFBoot and SH-aLRT suggesting that the relaxed trimming increased the signal to noise ratio in our dataset. However, despite the significant increase in the number of analyzed positions, the basal branches dividing archaeal and bacterial clades remained short, indicating a low rate of sequence evolution since the introduction of bacterial DNA gyrase in the archaeal domain.

Collectively, the data show that the bacterial and archaeal DNA gyrase sequences segregate into monophyletic clades that are consistently separated by short branches. Such tree topology is inconsistent with the presence of DNA gyrase in LUCA (the Last Universal Common Ancestor) or its early acquisition in the lineage leading to LACA, because such history should result in highly divergent proteins (Da Cunha et al. 2017; Catchpole and Forterre 2019; Coleman et al. 2021; Martinez-Gutierrez and Aylward 2021). Instead, our data are consistent with late emergence of DNA gyrase in the lineages leading to LACA or LBCA (Last Bacterial Common Ancestor) and transfer of DNA gyrase genes between the two domains just before or shortly after their diversification. Among different possible scenarios (see Discussion) the most parsimonious one suggests that DNA gyrase was introduced in an archaeal lineage by a single horizontal gene transfer from the ancestor of the Gracilicutes and/or the Terrabacteria.

### Phylogeny of Archaeal DNA Gyrases

**Fig. 4.**
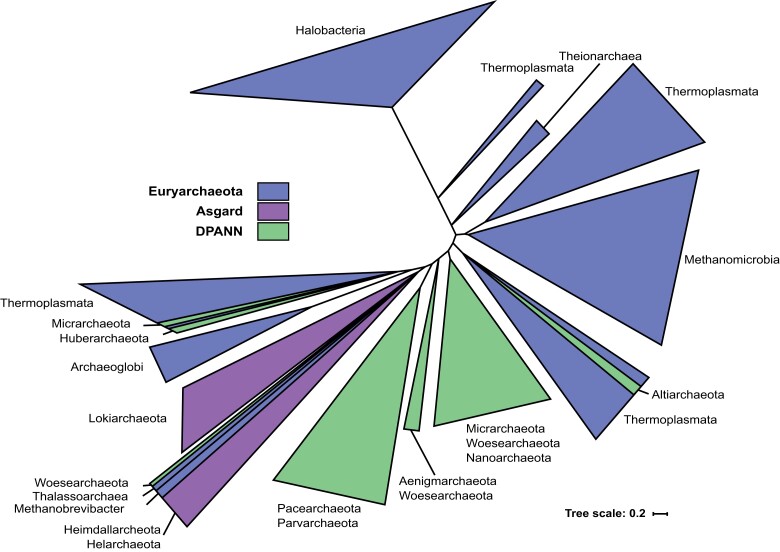
Vertical evolution is the predominant mechanism of gyrase spreading in the archaeal domain. Schematic representation of the maximum likelihood phylogeny of archaeal GyrA sequences. The detailed tree is shown in supplementary fig. S5, Supplementary Material online. The tree was inferred from the alignment of 377 GyrA sequences. The UFBoot support and SH-aLRT support are indicated for major bipartitions. Group I Euryarchaeota (Theionarchaea) are highlighted with an asterisk.

The global DNA gyrase tree indicated that the archaeal sequences were monophyletic but the internal topology of the Archaea was not robustly resolved. To increase the resolution, we performed a new phylogenetic analysis restricted to the archaeal DNA gyrases. The maximum likelihood trees inferred from the alignment of 377 GyrA or 331 GyrB sequences resolved well-supported clades corresponding to coherent taxonomic groups, except for the DPANN and Asgard superphyla (see supplementary figs S4 and S6, Supplementary Material online). A more relaxed alignment trimming produced a GyrA phylogeny in which the Asgard monophyly was recovered and the DPANN were distributed between three closely branching lineages (fig. 4, see supplementary fig. S5, Supplementary Material online). DNA gyrases from these two superphyla further branched within DNA gyrases of group II Euryarchaeota, such that this group was split into two clades, one including DNA gyrases from Asgard archaea and the other DNA gyrases from DPANN. In a few cases, DNA gyrases from a DPANN branched within the Asgard or Thermoplasmata clades, suggesting recent HGT events or alternatively artefactual branch attraction of these fast-evolving DPANN sequences. DNA gyrases are almost completely absent in group I Euryarchaeota, with the exception of a sporadic appearance in Theinoarchaea and *Methanobrevibacter*. DNA gyrases from Theinoarchaea form a monophyletic clade branching within group II Euryarchaeota and thus likely have acquired gyrase by a recent HGT. The position of *Methanobrevibacter* within the Asgard most likely results from an attraction by the long branches of Asgard DNA gyrases, since *Methanobrevibacter* DNA gyrases were recently acquired from bacteria (see fig. 3).

Although the use of a relaxed trimming of sequence alignments recovers a tree topology more congruent with the expected archaeal tree topology, branch support at some deep nodes remained weak thus resulting in a low resolution of the gyrase tree at the phylum level. These results are further difficult to interpret since there is no consensus regarding the rooting of the archaeal tree (Petitjean et al. 2015; Raymann et al. 2015; Da Cunha et al. 2017; Williams et al. 2017; Baker et al. 2020) and thus no true reference tree with which to compare. Nevertheless, the repeated recovery of monophyletic clades corresponding to major taxonomic divisions within archaeal superphyla is not consistent with the spread of DNA gyrase through multiple temporally separated HGT events. Rather, the data suggest predominantly vertical evolution with few early transfer events occurring before or at early stages of the diversification of the archaeal superphyla. Such evolution suggests that DNA gyrase was originally acquired by the lineage leading to group II Euryarchaeota (which all encode DNA gyrase) and introduced later to the Asgard and DPANN superphyla by secondary HGT.

### Potential Transfer Mechanisms for DNA Gyrase

The overall phylogeny of prokaryotic gyrases indicates that inter-domain HGT of genes encoding DNA gyrase is uncommon. This suggests that adaptation of an exogenous gyrase to a host cell from another domain is not trivial, even when this cell already encodes an endogenous gyrase. Still, at least one successful implantation of bacterial DNA gyrase in an archaeal cellular context must have occurred early in evolution. Closer examination of cases where functional gyrase genes were recently transferred between Archaea or from Archaea to Bacteria and *vice versa* could inform us about the necessary requirements for a successful transfer.

We first examined the possibility that DNA gyrase could travel between organisms *via* mobile genetic elements. A recent survey of 38,556 mobile genetic elements (ICE, plasmids and prophages) in Bacteria showed that *gyrA* genes are rare in these elements, indicating very limited use of these vehicles for the horizontal spread of DNA gyrase genes (Rodríguez-Beltrán et al. 2020). Using BLAST sequence similarity searches we performed a similar survey across the entire NCBI plasmid and virus databases (44,862 viruses and 31,939 plasmids, Sept. 2021) and did not recover significant *gyrA* hits originating from bacterial or archaeal plasmids. This indicates that plasmids do not typically transport DNA gyrase genes and suggests a different mechanism for the HGT of DNA gyrase genes into the archaeal domain.

In the case of viruses, we detected *gyrA*-like sequences in the genomes of 13 *Caudoviricetes* infecting *Bacillus* or *Lactococcus* bacteria. However, these sequences did not encode a GyrA box motif, suggesting that these are not functional DNA gyrase subunits (perhaps TopoIV, or degenerated gyrase subunits) (see supplementary fig. S7, Supplementary Material online). We also detected hundreds of *gyrA*-like sequences in metagenome-assembled genomes of bacterioviruses, all originating from one recent meta-analysis of human-associated viruses (Tisza and Buck 2021). These viruses were classified as *Microviridae* which have single-stranded DNA genomes, or *Myoviridae, Siphoviridae* and *Podoviridae* belonging to the class *Caudoviricetes* (head and tailed bacteriophages). The alignment of representative GyrA sequences for each of these taxa showed that DNA gyrase from *Caudoviricetes* contained the GyrA Box suggesting that these viruses may carry functional DNA gyrases (see supplementary fig. S8, Supplementary Material online).

Collectively, these data show that mobile genetic elements rarely, if at all, encode gyrases except perhaps *Caudoviricetes* from the human microbiome (and potentially other still unexplored microbiomes). This suggest that the dispersion of the DNA gyrase genes via these vehicles is not a common mechanism of transmission among organisms.

### Cases of Inter-Domain Horizontal Gene Transfer

**Fig. 5.**
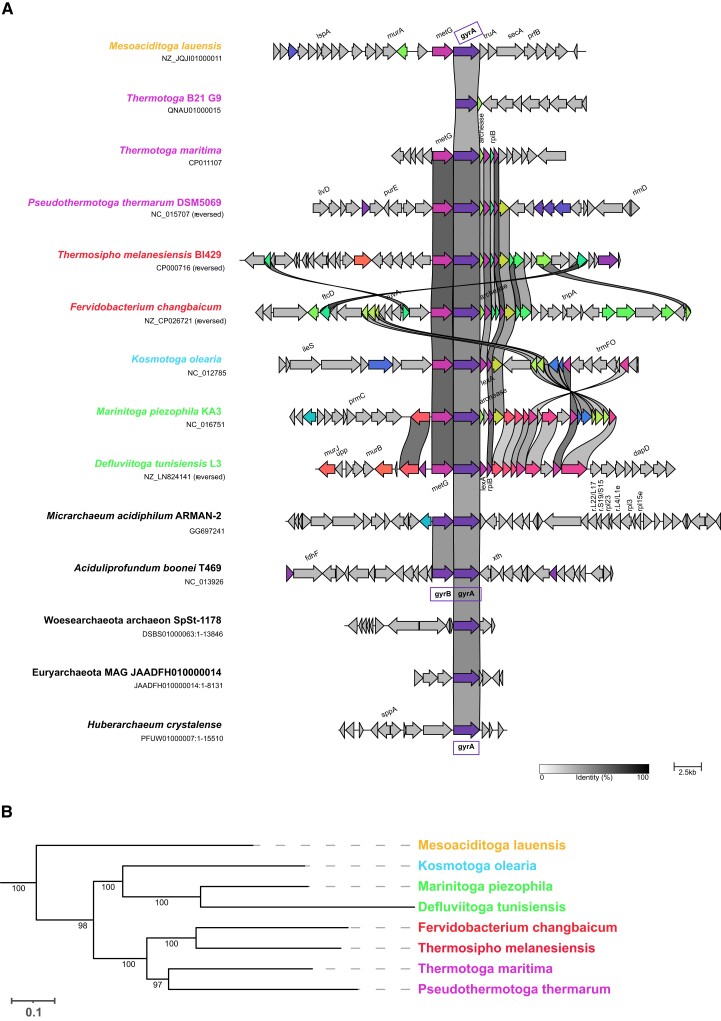
Synteny analysis of the *gyrA* locus in Thermotogales. (*A*) The genomic context around the *gyrA* gene is depicted with each arrow corresponding to a gene. Genes are automatically color-coded based on functional annotation. The scale bar at the bottom corresponds to the percentage of identity between proteins encoded by the depicted genes. The drawing is on scale with the scale bar representing 2.5 kb. The bacterial species names are indicated in color and the same color code is used for the phylogenetic tree shown on the right. (*B*) Phylogeny of Thermotogales species used in the synteny analysis. The phylogenetic tree was automatically generated using PhyloT and the Genome Taxonomy Database and visualized using iTOL. The numbers on the branches correspond to bootstrap node support values.

To gain further insight into the mode of transmission of DNA gyrase genes we examined the most robust cases of inter-domain HGTs apparent in our phylogenies—those of *Methanobrevibacter* and *Thermotoga* DNA gyrases (fig. 3). In global DNA gyrase phylogenies we consistently recovered *Methanobrevibacter* archaea within Firmicutes bacteria, and *Thermotoga* bacteria were positioned within a Thermoplasma/Archaeoglobi clade, in agreement with the thermophilic lifestyle of these organisms. In *Thermotoga*, the *gyrA* and *gyrB* genes are encoded by distant loci. For the *gyrA*-encoding locus, the extent of synteny correlates with the tree topology of Thermotogales, thus suggesting vertical inheritance of the DNA gyrase genes from the common ancestor of this group (fig. 5). However, the genomic context is not well conserved across different genera, suggesting a high degree of plasticity in this genomic region. Similar results were obtained for the *gyrB* locus (see supplementary fig. S9, Supplementary Material online). In the most closely related archaeal donor species, the DNA gyrase genes are organized in a *gyrBA* operon, indicating that this operon was disrupted after its transfer to Thermotogales.

**Fig. 6.**
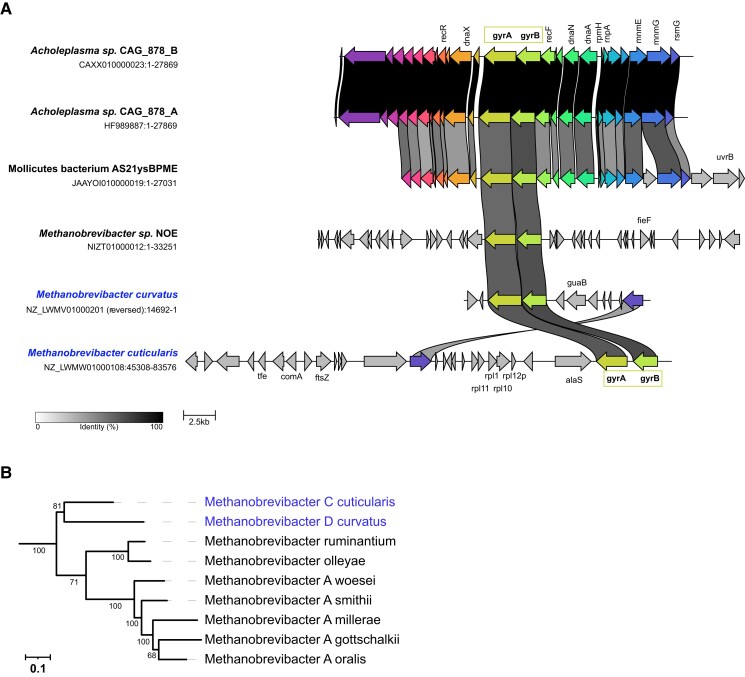
Synteny analysis of the *gyrBA* locus in *Methanobrevibacter* species. (*A*) The genomic contexts around *gyrBA* operons are depicted with each arrow corresponding to a gene. Genes are automatically color-coded based on functional annotation. The scale bar at the bottom corresponds to the percentage of identity between proteins encoded by the depicted genes. The drawing is on scale with the scale bar representing 2.5 kb. The two closely related *Methanobrevibacter* species are highlighted in blue. (*B*) Phylogeny of *Methanobrevibacter* species. The phylogenetic tree was automatically generated using PhyloT and the Genome Taxonomy Database and visualized using iTOL. The numbers on the branches correspond to bootstrap node support values.

The transfer of DNA gyrase genes from Firmicutes bacteria to *Methanobrevibacter* archaea must have been relatively recent since only few species of *Methanobrevibacter* carry DNA gyrase. As such, this example can inform us about the early stages of establishment of DNA gyrase in a naive organism. Synteny analysis showed that gyrase genes were clustered in *gyrBA* operons, however, there was no conservation of the surrounding genomic regions even between the closely related species *Methanobrevibacter curvatus* and *Methanobrevibacter cuticularis* (fig. 6). These two species are monophyletic, suggesting that DNA gyrase was introduced by a single transfer to their ancestor followed by genome rearrangements whereby the *gyrBA* operon was conserved. This suggests that the *gyrBA* operon arrangement may be under positive selection pressure; inactivation of DNA gyrase genes by genetic drift would be expected if DNA gyrase offers no selective advantage to *Methanobrevibacter* species. Using sequence alignment, we detected all catalytically important residues in these archaeal orthologs suggesting that these may indeed be functional (see supplementary fig. S10, Supplementary Material online). There was a single glycine to alanine mutation in a GyrA box motif which could potentially alter negative supercoiling activity. Together, the data suggest that DNA gyrase was successfully implanted in a few *Methanobrevibacter* species and seems to provide a selective advantage for these organisms. If functional, this could be used to develop genetic tools for these cultivable methanogens by using gyrase-targeting antibiotics.

### Co-evolution of DNA Gyrase and Topoisomerase VI in Archaea

DNA topoisomerase IB (Topo IB) and Topo VI are the only known archaeal topoisomerases that can relax positive supercoils *in vitro* (other than DNA gyrase). Based on this biochemical evidence and the almost ubiquitous distribution of Topo VI in Archaea, it was proposed that this enzyme is the ancestral archaeal type II DNA topoisomerase with an essential function in chromosome decatenation and the relaxation of positive supercoils (Forterre and Gadelle 2009). The introduction of a bacterial DNA gyrase into an ancestral archaeal cell would therefore generate a situation in which the gyrase activity would overlap with the primordial function of Topo VI. Despite this anticipated functional overlap, DNA gyrase has successfully established in many archaeal lineages alongside Topo VI, suggesting these enzymes have evolved toward specialized functions. Interestingly, these two enzymes co-localize in genomes of several archaea, perhaps suggesting a physical and/or functional interaction (Berthon et al. 2008).

**Fig. 7.**
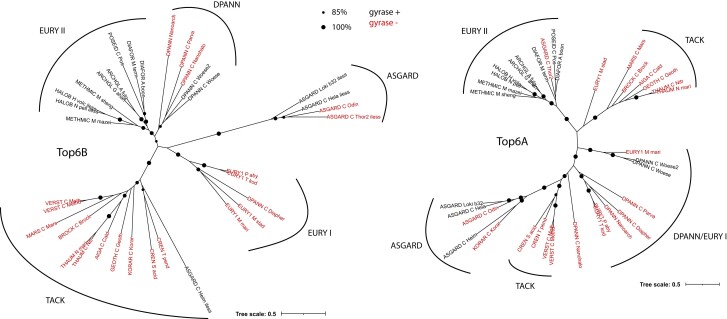
Phylogenetic tree of archaeal Top6A and Top6B orthologs. Selected sequences (*n* = 35) from DNA gyrase-less (red) and DNA gyrase-encoding (black) organisms were aligned using T-Coffee and non-homologous positions were removed using BMGE. The final alignment contained 420 positions for Top6B subunit and 332 positions for Top6A subunit. Maximum likelihood trees were constructed using IQ-tree with LG + F+I + G4 and LG + I+G4 sequence evolution models for Top6B and Top6A, respectively. Clades corresponding to four archaeal superphyla are indicated, note that Euryarchaeota was split into group I and group II Euryarchaeota as defined by Forterre et al. (2014).

As an *in silico* approach to testing this hypothesis, we compared the Topo VI sequences between gyrase-less and gyrase-encoding Archaea with the underlying hypothesis that such comparison may reveal lineage-specific or ancestral adaptations of Topo VI sequences to the presence of DNA gyrase as well as possible co-transfers. We first selected representative Topo VI sequences covering the entire archaeal taxonomic diversity and then built separate phylogenetic trees for subunit A (Top6A) and subunit B (Top6B). The resulting phylogenetic trees globally follow the classical archaeal taxonomy with the main phyla recovered as monophyletic clades (fig. 7). Importantly, Topo VI sequences from gyrase-encoding archaea did not cluster together, suggesting that independent, lineage-specific, rather than ancestral adaptations to DNA gyrase presence could have occurred in Archaea carrying DNA gyrase. This also indicates that Topo VI was never co-transferred with DNA gyrase despite co-localization of their genes in some Archaea.

**Fig. 8.**
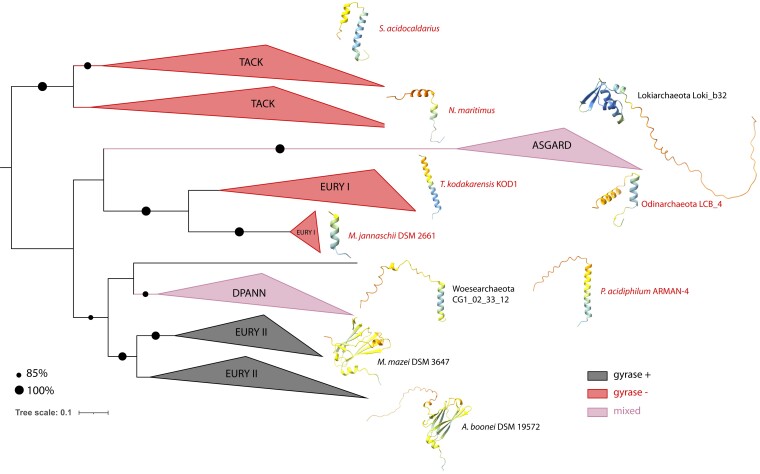
Structural diversity and phylogenetic distribution of C-terminal regions of archaeal Top6B orthologs. The phylogenetic tree of archaeal Top6B orthologs (Fig. 7) is shown with the rooting chosen arbitrarily. DNA gyrase-encoding clades are colored in red, clades lacking DNA gyrase are in black and mixed clades (with both gyrase-encoding and gyrase-less organisms) are colored in purple. AlphaFold2-predicted model structures of Top6B C-terminal extremities from representative organisms of each clade are shown. The structures are colored by the pLDDT confidence index (see also supplementary fig. 12, Supplementary Material online).

To explore this possibility further, we used the Top6B tree (which best fits the classical archaeal taxonomy) and generated separate sequence alignments for each of the five clades. This showed that most of the sequence variability between the clades occurred in the C-terminal region of Top6B (see supplementary fig. S11, Supplementary Material online). For example, the Top6B subunit of all Euryarchaeal group II organisms (containing gyrase) exhibit a C-terminal extension of about 100 residues which is absent in gyrase-less archaea. *De novo* structural modeling using AlphaFold2 revealed that this extension corresponds to a globular C-terminal domain (CTD) which adopts an immunoglobulin-like fold (Corbett et al. 2007) while the Top6B from gyrase-less TACK archaea contained a short C-terminal helix (see supplementary fig. S12, Supplementary Material online). Of note, the *de novo* AlphaFold2 prediction of Top6B structure from *Methanosarcina mazei* (for which an X-ray crystallographic structure is available) was globally accurate including the CTD domain (see supplementary fig. S12*B*, Supplementary Material online). To determine if a particular C-terminal extension of Topo VI is correlated with the presence of DNA gyrase, we extended our AlphaFold2 modeling to representative sequences across the entire Top6B phylogenetic tree (fig. 8, see supplementary fig. S12, Supplementary Material online). This analysis revealed that the C-terminal region of Topo VI (following the transducer domain) in DNA gyrase-less organisms is systematically modeled as one or two alpha helices, whereas DNA gyrase-encoding organisms (Asgard and Euryarchaea group II) do not seem to contain similar alpha helices, instead encoding a globular domain.

Taken together, the combination of phylogenetic and structural analyzes suggests the existence of lineage-specific C-terminal extensions within Top6B sequences of DNA gyrase-encoding archaea while the Topo6B of DNA gyrase-less archaea sampled from phylogenetically distant phyla all possess shorter and structurally similar C-terminal architectures. This suggests the existence of at least three independent yet convergent pathways for adaptation of TopoVI sequences following the acquisition of gyrase in Euryarchaeal group II, DPANN and Asgard lineages.

## Discussion

DNA topoisomerases are ancient enzymes with a complex evolutionary history. These ubiquitous enzymes must have played an important but still poorly understood role in the evolution of genomes. In this study, we focused on the evolutionary history of DNA gyrase, a type II topoisomerase which is ubiquitous in Bacteria but has a patchy distribution in Archaea.

### Evolution of Archaeal DNA Gyrases

**Fig. 9.**
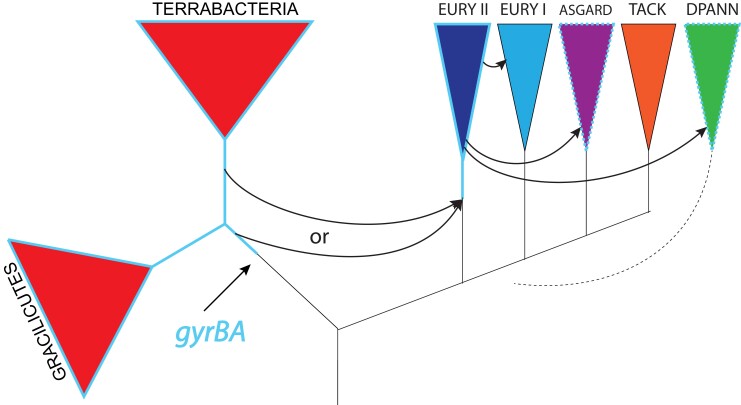
Proposed scenario for emergence and spread of DNA gyrase in Archaea. DNA gyrase emerged in a bacterial lineage and was transferred once to the lineage leading to group II Euryarchaeota. The donor organism was a bacterium from the lineage which will give rise to Gracilicutes and/or Terrabacteria. From group II Euryarchaeota DNA gyrase was transferred to DPANN and Asgard groups early during the diversification of these lineages via rare HGT events and, more recently, to few Theionarchaea (Euryarchaeota I). In this scenario, LACA is older than LBCA (if the donor was pre-LBCA bacterium) or LACA is older than the last common ancestor of Terrabacteria (or Gracilicutes). Black arrows symbolize horizontal gene transfer events. Blue lines indicate presence of DNA gyrase in a lineage or a group. Dotted blue line indicates partial presence of DNA gyrase in a lineage or group. The dotted branch symbolizes the uncertain position of DPANN superphylum within the archaeal tree.

An important observation arising from our phylogeny of DNA gyrases is that the bacterial and archaeal clades are consistently separated by short branches. A similar result was previously obtained with the phylogenetic analyzes of another DNA topoisomerase, reverse gyrase (Catchpole and Forterre 2019). These two domains are typically separated by long branches in phylogenies of universal marker proteins (Da Cunha et al. 2017; Catchpole and Forterre 2019; Coleman et al. 2021; Martinez-Gutierrez and Aylward 2021), suggestive of a fast rate of sequence evolution between LUCA and the respective ancestors of Bacteria and Archaea, resulting in very divergent proteins (Woese 1998; Forterre 2006). Consequently, our phylogenetic analysis strongly suggests that DNA gyrase was not present in LUCA but was acquired shortly before or after diversification of Bacteria or Archaea. This implies its present distribution results from a transfer from Bacteria to Archaea or *vice versa*. Both hypotheses are equally valid if the root of the archaeal tree is located between group I and II Euryarchaeota (Raymann et al., 2015, Aouad et al. 2022). However, considering the restricted distribution of DNA gyrase in Archaea and its ubiquity in Bacteria, it seems more parsimonious to imagine that DNA gyrase was already present in the LBCA and was transferred later to the ancestor of the group II Euryarchaeota. In such a case, the donor would be an ancient bacterium from the lineage leading either to the Gracilicutes and/or the Terrabacteria. This first transfer event would then be followed by spread of DNA gyrase to subsets of DPANN and Asgard lineages via secondary transfers (fig. 9). The paraphyletic nature of group II Euryarchaeota in the archaeal DNA gyrase tree (fig. 4) indicates that these secondary transfers occurred shortly after the diversification of group II Euryarchaeota. This scenario is also the most compatible with the rooting of the Archaea between Euryarchaeota and all other archaeal phyla (Petitjean et al. 2015), or in the branches leading either to DPANN archaea (Williams et al. 2017; Baker et al. 2020) or to the proposed BAT superphylum (Bathyarchaea, Aigarchaeoa and Thaumarchaea) (Da Cunha et al. 2017). Regardless of the rooting of the archaeal tree, a transfer from Archaea to Bacteria would require independent losses of DNA gyrase genes in all archaeal lineages to explain its present-day distribution. However, the systematic presence of DNA gyrase in all members of group II Euryarchaeota suggests this enzyme cannot be lost once it has been introduced in a lineage (in agreement with experimental data obtained in halobacteria and methanogens).

### Gyrase Phylogeny Resolves the Deepest Phylogenetic Divide within the Bacterial Domain

A recent study used information from gene duplications and losses as well as gene transfers between genomes to root the bacterial tree without including an archaeal outgroup (Coleman et al. 2021). This approach resulted in division of bacteria into Terrabacteria (a clade encompassing Cyanobacteria, *Deinococcus/Thermus*, Firmicutes, etc.) and Gracilicutes (a clade encompassing Proteobacteria, Bacteroidetes, Spirochetes, etc.) corresponding to the deepest phylogenetic divide within the bacterial domain. Remarkably, we recovered this earliest divergence in the global DNA gyrase tree, suggesting that DNA gyrase may be a suitable marker for investigating deep phylogenetic relationship between bacterial lineages using archaeal orthologs as outgroup without undesired long branch attraction effects. An interesting perspective would be to search for other genes with a similar evolutionary history (origin in an ancestor of one domain and late gene transfer to the other domain) with a short branch separating the two domains to help resolve the earliest events during the evolution of life.

The analysis of ancestral HGT can also provide insight into the relative age of taxonomic groups, because the donor organism must be at least as old as the recipient. According to the most parsimonious scenario (fig. 9), LACA is older than ancestor of Terrabacteria (or Gracilicutes) and possibly even older than LBCA.

### Why is DNA Gyrase not more Widely Spread in Archaea?

The similar level of DNA gyrase sequence conservation in Archaea and Bacteria (fig. 1) suggests that the DNA gyrase genes did not require significant changes to adapt to the archaeal cellular context. In line with this observation, we have recently successfully expressed bacterial DNA gyrase in the archaeon *Thermococcus kodakarensis* (Villain et al. 2021). Remarkably, the DNA gyrase activity flipped the DNA supercoiling of this archaeon from positive to negative without a measurable effect on growth, suggesting that this organism is highly resilient with regard to DNA topology. This begs the question as to why DNA gyrase has not been transferred more frequently from Bacteria to Archaea or within Archaea, if such transfers are easily tolerated. One explanation could be the rarity of mobile elements encoding DNA gyrase genes. Indeed, in contrast to the situation previously observed with DNA topoisomerases members of the Topo IIB family ((Takahashi et al. 2020) and this work) we did not find conjugative plasmids or viruses encoding DNA gyrase in Archaea. We only detected two robust cases of relatively recent HGT between Archaea and Bacteria. In both cases, the putative donor organism encoded DNA gyrase genes as a *gyrBA* operon (figs 5 and 6). This makes sense since the co-localization of genes within an operon facilitates their transfer by HGT and both genes are required to form functional tetrameric A_2_B_2_ holoenzyme. In Thermotogales, however, the operon structure is split into two distant genomic loci and a similar situation is found in many bacterial and archaeal lineages (data not shown) indicating, at least in these lineages, the absence of selective pressure for the maintenance of an operon structure. Since organisms bearing clustered genes are more likely to act as successful donors (Lawrence and Roth 1996), the separation of DNA gyrase genes into two distant genetic loci might be another reason limiting HGT among bacterial and archaeal genomes.

Interestingly, organisms from donor and recipient clades of recent HGT events were isolated from the same environments. This is the case for the *Thermotoga*/*Archaeoglobus* pair which inhabit deep-sea hydrothermal vents (Wery et al. 2002); for the Ca. *Micrarchaeum acidiphilum* ARMAN2/*Thermoplasma* (Baker et al. 2010) pair found in acidophilic ecosystems; and for the *Methanobrevibacter*/Firmicutes pair, living in close contact in termite intestinal tracts (Leadbetter and Breznak 1996). It seems, therefore, that physical proximity is required for the successful HGT of DNA gyrase genes. Interestingly, DPANN organisms are obligatory symbionts/parasites living in physical contact with the surface of their hosts, and exchange of genes with their hosts has been already documented (Sakai et al. 2022). Similarly, the only cultivated Asgard archaeon lives in symbiosis with a methanogen belonging to group II Euryarchaeota (Imachi et al. 2020). The existence of a similar relationship between the ancestors of Asgard and DPANN archaea and Euryarchaeal group II may explain why DNA gyrase spread into these lineages.

### Histones as Facilitating Factor for Gyrase Emergence in Archaea?

The first recipient of DNA gyrase was probably an ancestor of the Euryarchaeal group II indicating that its “topological kit” was compatible with the presence of DNA gyrase. Compilation of data from published phylogenomics studies (Forterre and Gadelle 2009; Groussin and Gouy 2011; Soppa 2011; Raymann et al. 2014; Peeters et al. 2015) revealed that the putative ancestor was equipped with one type II topoisomerase enzyme (Topo VI), two type I topoisomerases (Topo III and Reverse gyrase), two nucleoid associated proteins (Alba and TrmBL2), one SMC (Structural maintenance of chromosomes) protein and two histones. The same topological kit is present in the hyperthermophilic archaeon *T. kodakarensis* in which we successfully expressed bacterial DNA gyrase genes (Villain et al. 2021). Notably, transcriptomic analysis has shown that *T. kodakarensis* cells containing DNA gyrase did not overexpress endogenous DNA topoisomerases to counteract the negative supercoiling introduced by this enzyme (Villain et al. 2021). Archaeal and eukaryotic nucleosomes were reported to have the capacity to wrap DNA positively (Musgrave et al. 2000; Furuyama and Henikoff 2009), we thus proposed that the plectonemic negative supercoils can be efficiently removed by the positive supercoiling of DNA around nucleosomes. Moreover, all Archaea harboring a DNA gyrase also contain histones (with notable exception of *Thermoplasma* species), the presence of histones may thus be a prerequisite for DNA gyrase adaptation in Archaea. It would be interesting to test this hypothesis by expressing DNA gyrase in a histone-less archaeon.

### Co-evolution of DNA Gyrase and Topo VI in Archaea

The Archaea containing DNA gyrase from group II Euryarchaeota have negatively supercoiled plasmids and DNA gyrase is essential for these organisms, with an active (albeit poorly understood) role in modulating DNA supercoiling *in vivo* (Holmes and Dyall-Smith 1991; Yang et al. 1996; Sioud et al. 1988b; Tarasov et al. 2011). Our analysis now shows that DNA gyrases from DPANN and Asgard archaea also carry the canonical GyrA box motif and catalytic residues, suggesting that DNA gyrase can introduce negative supercoils in the genomes of these organisms. This suggests that the mechanisms dealing with DNA have adapted to the presence of DNA gyrase and became dependent on negative supercoiling in all Archaea containing this enzyme. Hence, a number of gene-specific and/or genome-wide adaptations may have occurred since the acquisition of DNA gyrase by Archaea. To explore this further, we focused on the Topo VI enzymes which are the ancestral archaeal type II DNA topoisomerases with essential functions seemingly overlapping with those of DNA gyrase. Remarkably, in *Thermoplasma*, DNA gyrase has replaced Topo VI as the only type II topoisomerase, thus demonstrating that DNA gyrase can accomplish the canonical functions of Topo VI alone (Kawashima et al. 2000). In halophilic and some methanogenic archaea, the genes encoding DNA gyrase and Topo VI are contiguous and are likely transcribed from divergent promoters (Berthon et al. 2008) thus further reinforcing the idea that Topo VI and DNA gyrase activities must be coordinated. Corbett and colleagues first hypothesized that Topo VI may be “sequestered” in a particular subcellular region in Euryarchaeal group II via an immunoglobulin-like fold domain found at the C-terminus of Top6B (Corbett et al. 2007). We found indeed such C-terminal extensions in DNA gyrase-encoding DPANN and Asgard archaea but never in DNA gyrase-less archaea, thus corroborating the idea that DNA gyrase introduction in Archaea triggered the evolution of Topo VI towards specialization to avoid interference with DNA gyrase. In agreement with this hypothesis, a recent study demonstrated that *M. mazei* Topo VI is preferentially a decatenase, suggesting that in this organism Topo VI functions in decatenation of interlinked chromosomes while DNA gyrase controls the overall DNA topology by introducing negative supercoils and by relaxing positive supercoils introduced during DNA replication and transcription (McKie et al. 2022).

Collectively, our study reveals evolutionary history of gyrase in Archaea and provides testable hypotheses to understand the prerequisites for successful establishment of gyrase in a naïve archaeon and the associated adaptations in the management of topological constraints.

## Materials and Methods

### Generation of the DNA Gyrase Data Set

GyrA and GyrB archaeal data sets were constructed by querying the National Center for Biotechnology Information (NCBI) data bases, for *Escherichia coli* GyrA (EGT66353.1) and GyrB (AKE86808.1) homologs using the BlastP program (Altschul et al. 1997). Additional searches were performed in 368 metagenome assemblies of Asgard genomes (as of November 2021) and Verstraetearchaeota genomes by tBlastN. Only sequences containing between 450 and 1,100 amino acids and sharing at least 25% sequence identity and >30% of query sequence coverage were selected. To detect inteins, GyrA and GyrB datasets were aligned separately and visualized using Geneious 11.0.5 (Biomatters), allowing the identification of 23 GyrA and 61 GyrB intein-containing sequences which were removed from the dataset. Using this approach, as of 5th of March 2020, 741 archaeal GyrA and 719 archaeal GyrB were retrieved. For bacterial GyrA and GyrB orthologs, the Annotree database was searched using KEGG accession numbers for GyrA (K02469) and GyrB (K02470) (Mendler et al. 2019). The 25,877 results for GyrA and 25,890 results for GyrB were downloaded. To avoid contamination of the GyrA dataset by topoisomerase IV (Topo IV) A subunits, sequences lacking the GyrA box motif Q(R/K)RGG(R/K)G were eliminated. Dataset complexity was reduced by removing sequences with >90% sequence similarity across 70% of the sequence length for archaeal sequences and 70% sequence similarity across 70% of the sequence length for bacterial sequences using MMseqs2 v11.e1a1c (Steinegger and Söding 2017). The final datasets for construction of phylogenetic trees contained 377 GyrA and 331 GyrB archaeal sequences and 799 GyrBA concatenated sequences (502 bacterial and 297 archaeal sequences). All sequence datasets used in this study were deposited using the figshare website and are available under DOI: 10.6084/m9.figshare.19137899.

### Analysis of Sequence Conservation

Bacterial and archaeal sequence datasets were compared using all against all BLASTp analysis (*E*-value <0.001). The median value for sequence identity and dispersion around this value was calculated using R 3.6.1 and plotted using the ggplot2 package with geom_violin function (https://CRAN.R-project.org/package=ggplot2).

The ConSurf server (http://consurf.tau.ac.il/) (Ashkenazy et al. 2016) was utilized to identify domain-specific conserved sites in the DNA gyrase dataset. The analysis was conducted using a Bayesian procedure and *E. coli* DNA gyrase (PDB: 6RKW) as template.

### Phylogenetic Tree Construction

Sequence alignment and trimming were performed using the Galaxy web platform (Afgan et al. 2018). GyrA and GyrB datasets were aligned with MAFFT v7.273.1 (Katoh and Standley 2013) using BLOSUM30 matrix. Selection of phylogenetically informative positions in alignments was done using BMGE v1.12 (Criscuolo and Gribaldo 2010) with the following parameters: BLOSUM30 matrix, sliding windows size 3, minimum block size 1. This filtering method removed 84% of positions (1288 remaining positions out of 7978). In parallel, less strict trimming was performed with Noisy (Dress et al. 2008) using 0.6 cut-off value to test for artificial shortening of branch lengths (Tan et al. 2015). Trimming the alignment of the complete DNA gyrase dataset with this method resulted in removal of 60% of positions (3,217 remaining positions out of 7,978). Trees were generated using IQ-TREE multicore v1.6.7 (Nguyen et al. 2015) on the Genotoul computing cluster (http://bioinfo.genotoul.fr/). Branch support was assessed using ultrafast bootstrap method (UFBoot, 1,000 iterations) (Hoang et al. 2018) and Shimodaira-Hasegawa approximate likelihood ratio test (SH-aLRT, 1,000 iterations) (Guindon et al. 2010). Typically, clades are considered reliable if SH-aLRT ≥80% and UFboot ≥95%. Automatic sequence evolution model selection was performed using ModelFinder with –m MFP option (Kalyaanamoorthy et al. 2017). Tests of tree topology were performed in IQ-TREE with 10,000 resamplings using the RELL method.

Trees were visualized using FigTree v1.4.4 (http://tree.bio.ed.ac.uk/software/figtree/). We systematically investigated the genomic context of highly divergent sequences (appearing as long branches in the trees) or sequences branching outside their taxonomic group to detect contamination with misannotated or outlier fast-evolving sequences. Sequences from small contigs (<10 kb) or from contigs carrying rRNA genes not congruent with their annotation were removed from the data set. The dataset used for building final phylogenetic trees were deposited at the figshare website (DOI: 10.6084/m9.figshare.19137899).

For building a concatenated GyrA_GyrB tree, we first confirmed the presence of both *gyrA* and *gyrB* genes within the same genomes using Taxonomy ID. The two proteins were aligned using MAFFT, concatenated, and we performed the phylogenetic analysis using IQ-tree with the the MPF option for sequence evolution model selection.

### Synteny Analysis

The synteny conservation analysis around the *gyrBA* locus was performed using the Clinker tool (Gilchrist et al. 2021). We selected organisms for which we detected, based on our phylogenetic analysis, putative HGT of gyrase genes. We extracted the corresponding GenBank file containing the contig encoding the *gyrA* and or the *gyrB* genes or a 30 kb window of the chromosome or contig (if >50 kb). Global comparative analysis of our selected set of GenBank files was done using default parameters. The gene clusters were determined on the basis of protein similarity and the color-coded graphics were plotted.

### Topoisomerase VI Sequence and Phylogenetic Analysis

Top6A and Top6B sequences were recovered from the UniProt database or by BLASTp searches. Several sequences contained inteins that we removed manually. The curated set of Top6A and Top6B sequences (*n* = 35) was aligned separately using T-coffee web server (Di Tommaso et al. 2011) with default settings. The obtained alignments were trimmed using BMGE v1.12 with the following parameters: BLOSUM30 matrix, sliding windows size 3, minimum block size 3. Secondary structure information was rendered using ESPript 3.0 (Robert and Gouet 2014) and the sequence of Topo VI from *M. mazei* (PDB 2q2e) as reference. Maximum likelihood tree construction was done with IQ-TREE and branch support was assessed using UFboot with 1000 replicates and the trees were edited using iTOL (Letunic and Bork 2021). AlphaFold2 (Jumper et al. 2021) batch colab (https://colab.research.google.com/github/sokrypton/ColabFold/blob/main/batch/AlphaFold2_batch.ipynb) was used to predict de novo structures of representative Top6B sequences. Model structures were visualized and edited with UCSF ChimeraX v. 1.3 (Pettersen et al. 2021).

## Supplementary Material

msac155_Supplementary_Data

## Data Availability

All datasets used in this study were deposited using the figshare website and are available under DOI: 10.6084/m9.figshare.19137899.
