# Supplementary material for: Expanded Dataset Reveals the Emergence and Evolution of DNA Gyrase in Archaea
Source: Mol Biol Evol. 2022 Jul 9;39(8):msac155. doi: 10.1093/molbev/msac155 (PMC9348778; doi:10.1093/molbev/msac155)
Supplement: msac155_Supplementary_Data [file msac155_supplementary_data.pdf]

SUPPLEMENTARY FIGURES AND TABLES

Supplementary table 1. Dispersion of sequence identity values for prokaryotic GyrA and GyrB sequences

		**Min.***	**1st Qu.**	**Median**	**Mean**	**3rd Qu.**	**Max.**
**GyrA**	Bacteria	16.67	36.75	43.55	41.11	47.40	100
	Archaea	16.79	28.14	46.17	43.67	51.02	100
	Archaea vs Bacteria	15.96	30.61	44.30	40.72	47.78	95.68
**GyrB**	Bacteria	21.12	45.57	49.75	50.21	54.36	100
	Archaea	27.18	52.38	55.99	57.25	59.62	100
	Archaea vs Bacteria	23.12	48.62	52.60	52.57	56.52	96.26

*All values are given as percentage

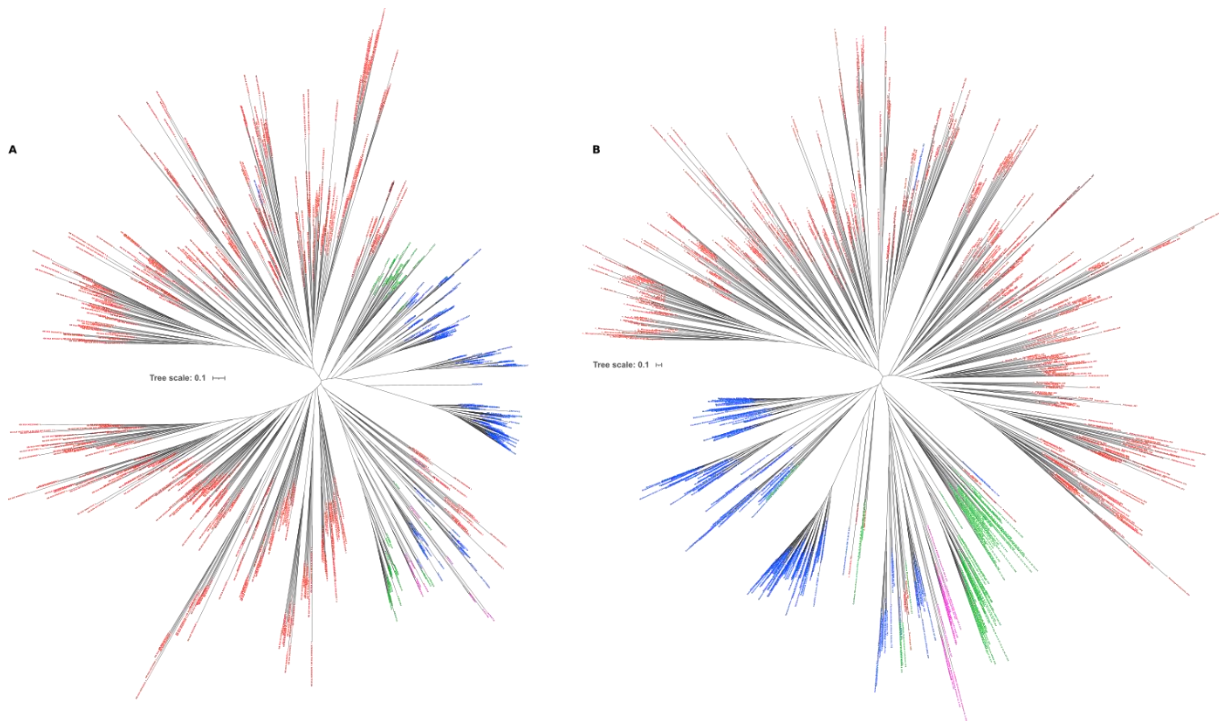

Supplementary figure 1. Global phylogeny of DNA gyrases

The GyrA and GyrB (502 bacterial and 297 archaeal) sequences were concatenated, aligned using MAFFT and trimmed using BMGE (A) or Noisy (B). This operation removed 6690 out of 7978 positions from the alignment when using BMGE and 4761 out of 7978 positions when using Noisy. Maximum-likelihood analysis was used for tree construction with IQ-TREE and branch support was assessed using UFboot and SH-aLRT (1000 iterations). The consensus trees are shown where sequences belonging to Euryarchaea, DPANN and Asgard superphyla are coloured in blue, green and magenta, respectively. Bacteria are coloured in red. The tree scale corresponds to number of substitutions per site.

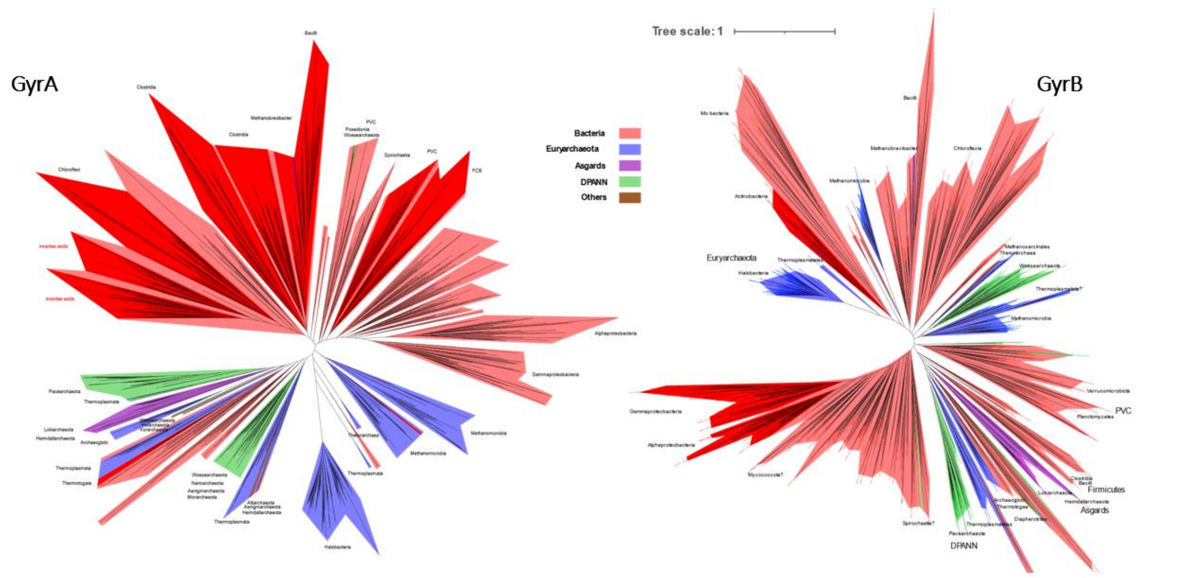

Supplementary figure 2. Global phylogeny of GyrA or GyrB sequences inferred with stringent sequence alignment trimming

The GyrA (571 bacterial and 385 archaeal) or GyrB (633 bacterial and 348 archaeal) sequences were aligned using MAFFT and trimmed using BMGE. This operation removed 359 out of 1085 and 528 out of 1099 positions for GyrA and GyrB alignments, respectively. Maximum-likelihood analysis was used for tree construction with IQ-TREE and branch support was assessed using UFboot and SH-aLRT (1000 iterations). The consensus trees are shown where bacterial sequences are underlined in red. The legend indicates color-codes for the archaeal superphyla. The tree scale corresponding to number of substitutions per site is indicated.

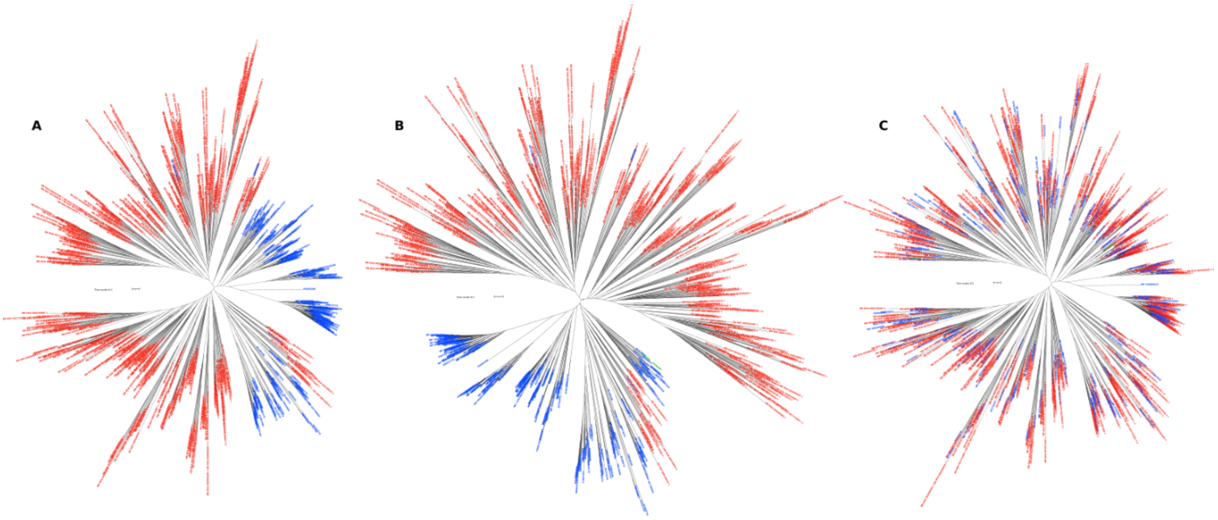

	bp-RELL	p-KH	p-SH	p-WKH	p-WSH	c-ELW	p-AU
Tree A	0.551 +	0.554 +	1	0.554 +	0.802 +	0.549 +	0.561 +
Tree B	0.449 +	0.446 +	0.72 +	0.446 +	0.692 +	0.451 +	0.439 +
Tree C	0 -	0 -	0 -	0 -	0 -	0 -	2.61e-78 -

Supplementary figure 3. Global gyrase tree topologies used for tests of tree selection.

A) Complete gyrase phylogeny inferred from BMGE-trimmed alignment. B) The complete gyrase phylogeny where one deep branch was moved to produce monophyletic archaeal clade. C) Complete gyrase phylogeny where the branches were shuffled but the global tree topology was preserved. Results of tree selection tests are shown on the bottom part of the figure. Values indicate p-value for exclusion and are colored in green for tree topologies not excluded by the tests, red for tree topologies significantly excluded. KH is one-sided Kishino-Hasegawa test; SH is Shimodaira-Hasegawa test; WKH is weighted KH test; WSH is weighted SH test; ELW is Expected Likelihood Weight; AU is approximately unbiased test. Plus signs denote the 95% confidence sets. Minus signs denote significant exclusion. All tests were performed with 10000 resamplings using the RELL method.

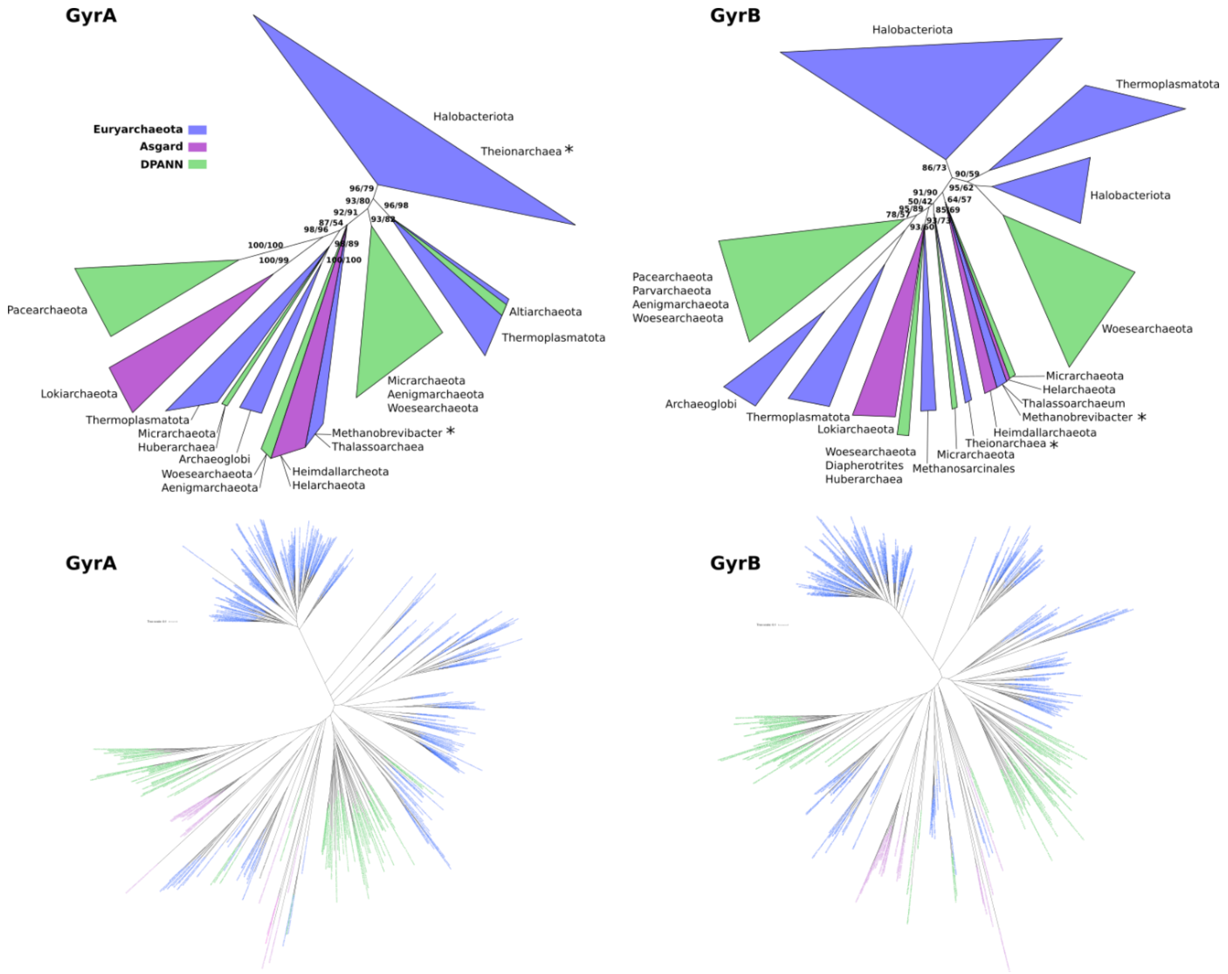

Supplementary Figure 4. Maximum likelihood phylogeny of archaeal GyrA or GyrB sequences inferred from conservatively trimmed sequence alignment.

376 GyrA and 331 GyrB sequences were aligned using MAFFT and trimmed using BMGE (see material and methods for details). The filtering removed 48 % of positions (779 from 1493 positions left) and 60% of positions (614 from 1554 left) for GyrA and GyrB alignment, respectively. Maximum-likelihood analysis was used for tree construction with IQ-TREE and branch support was assessed using UFboot and SH-aLRT (1000 iterations). The consensus trees are shown where sequences belonging to Euryarchaea, DPANN and Asgard superphyla are coloured in blue, green and magenta, respectively. The detailed trees are shown in the lower panel and their schematic representation is shown in the upper panel. The numbers on the branches indicate their UFboot and SH-aLRT support. Group I Euryarchaeota are indicated with an asterisk and the number of sequences present is indicated in brackets. The tree scale corresponding to number of substitutions per site is indicated.

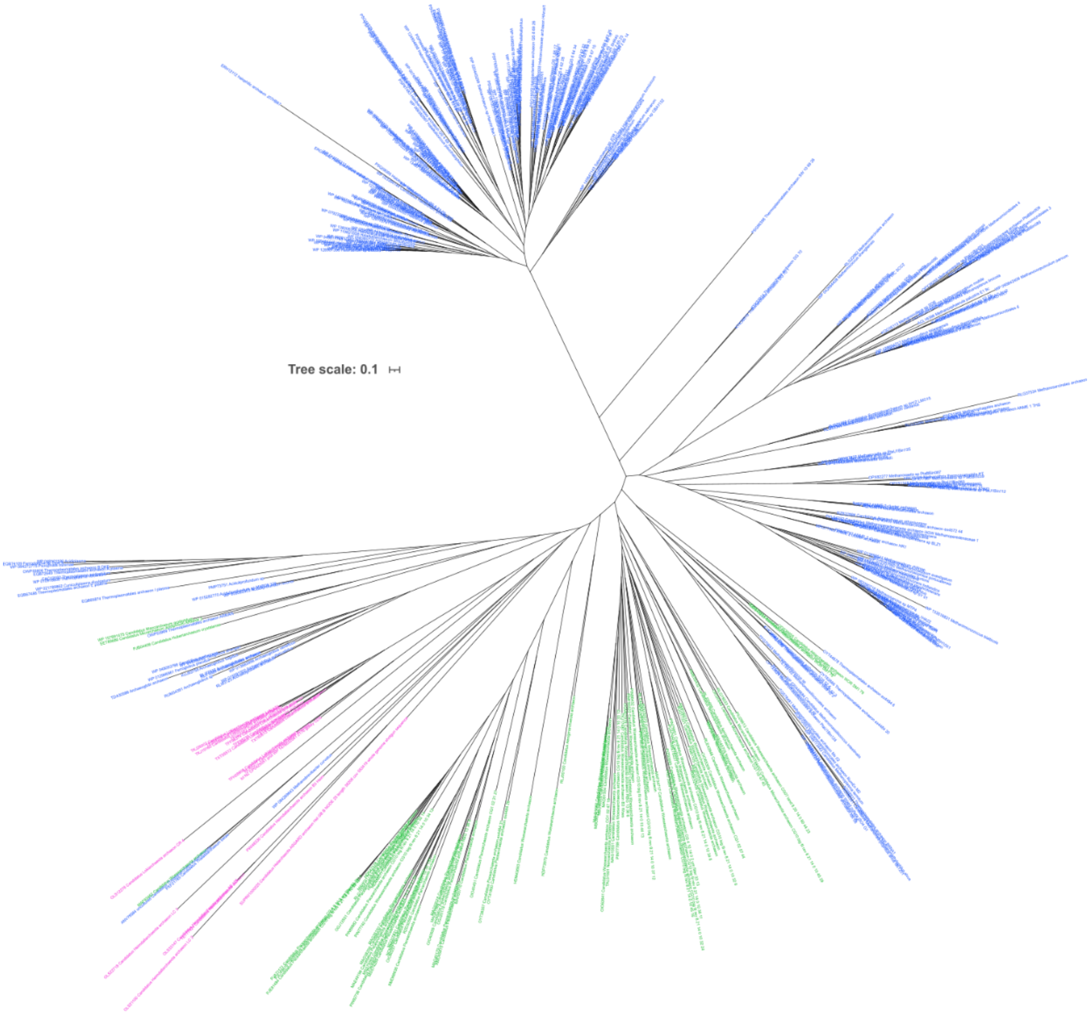

Supplementary figure 5. Maximum likelihood phylogeny of archaeal GyrA sequences.

Dataset containing 376 GyrA sequences was aligned using MAFFT and the phylogenetically uninformative regions were removed using Noisy. 1218 positions out of 1504 were used to produce the phylogenetic tree. The consensus trees is shown where sequences belonging to Euryarchaeota, DPANN and Asgard superphyla are coloured in blue, green and magenta, respectively. The tree scale corresponding to number of substitutions per site is indicated.

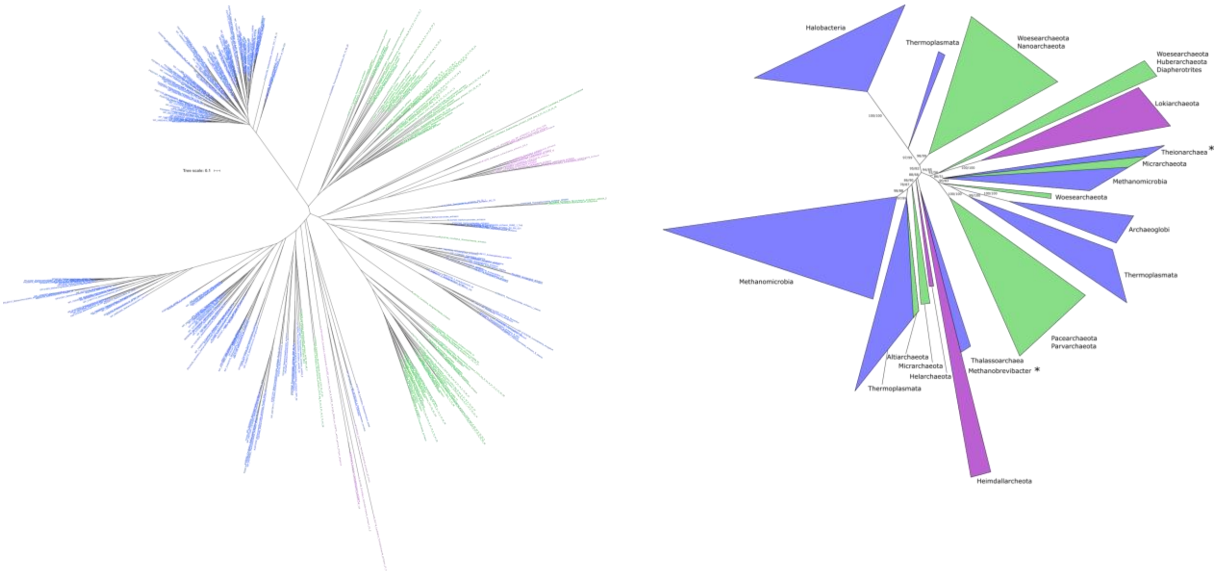

Supplementary figure 6. Maximum likelihood phylogeny of archaeal GyrB sequences.

Dataset containing 331 GyrB sequences was aligned using MAFFT and the phylogenetically uninformative regions were removed using Noisy. 826 positions out of 1551 were used to infer the phylogenetic tree. Detailed consensus tree is shown on the left, sequences belonging to Euryarchaeota, DPANN and Asgard superphyla are coloured in blue, green and magenta, respectively. The tree scale corresponding to number of substitutions per site is indicated. Schematic representation of GyrB tree is shown on the right. The members of group I Euryarchaeota are indicated with an asterisk. The numbers correspond to SH-aLRT and UFboot support for the deep nodes

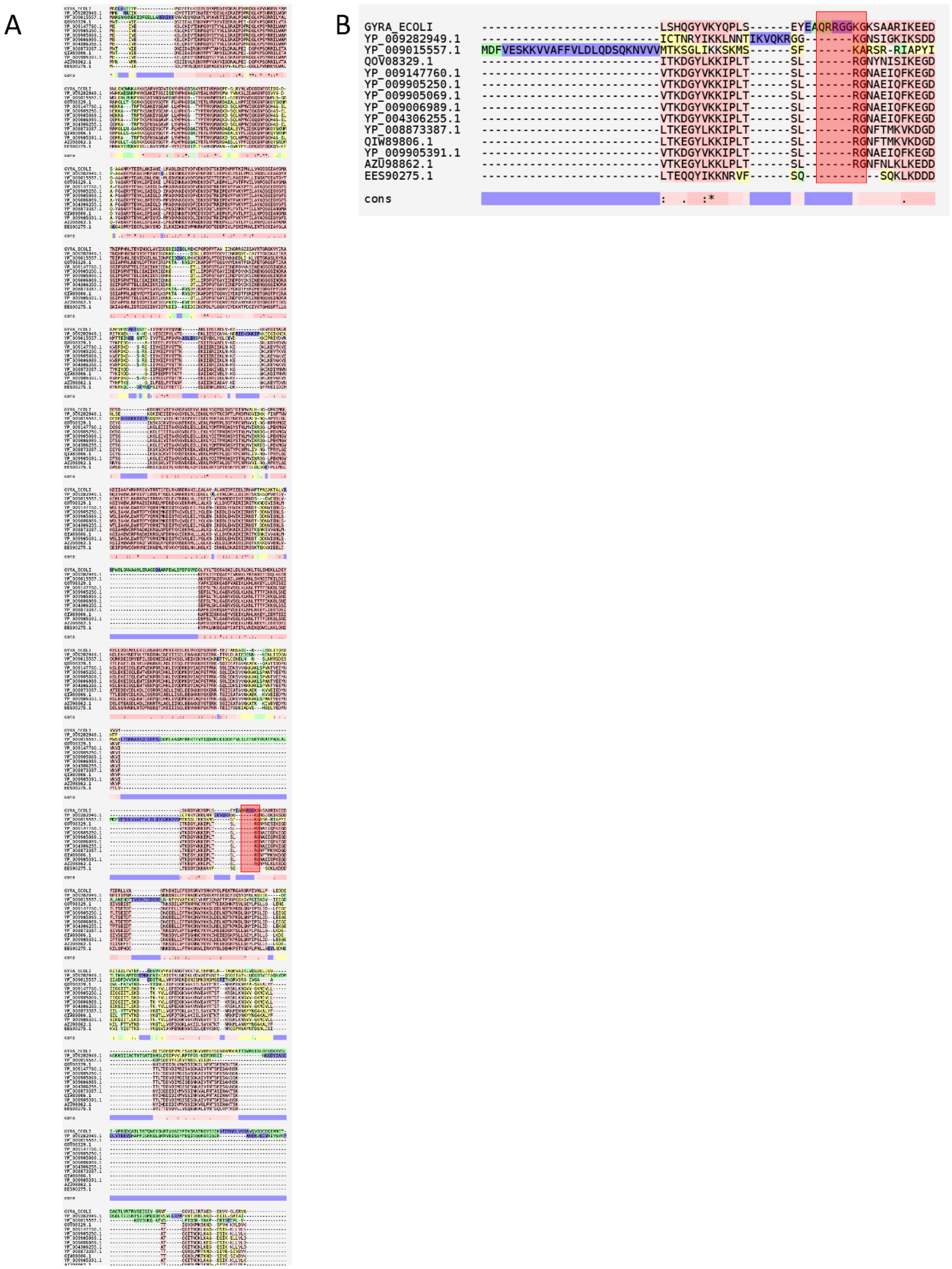

Supplementary figure 7. Alignment of GyrA-like sequences from isolated bacteriophages.

BLASTp search was used to identify viral homologs of *E. coli* GyrA (Uniprot ID: P0AES4). Viral sequences showing more than 25 % sequence identity over at least 70 % of sequence length were selected. Those sequences corresponded to bacteriohages infecting *Bacillus* or *Lactococcus* bacteria. Alignment was performed using T-coffee web server (Di Tommaso et al. 2011) with default settings.

Part A shows the global alignment with GyrA box (QRRGGKG) highlighted with a red rectangle and part B is enlarged part of the alignment containing the GyrA box. The first sequence is GyrA of *E. coli* which serves as a reference for canonical GyrA protein. The alignment shows that viral sequences do not contain GyrA box motif.

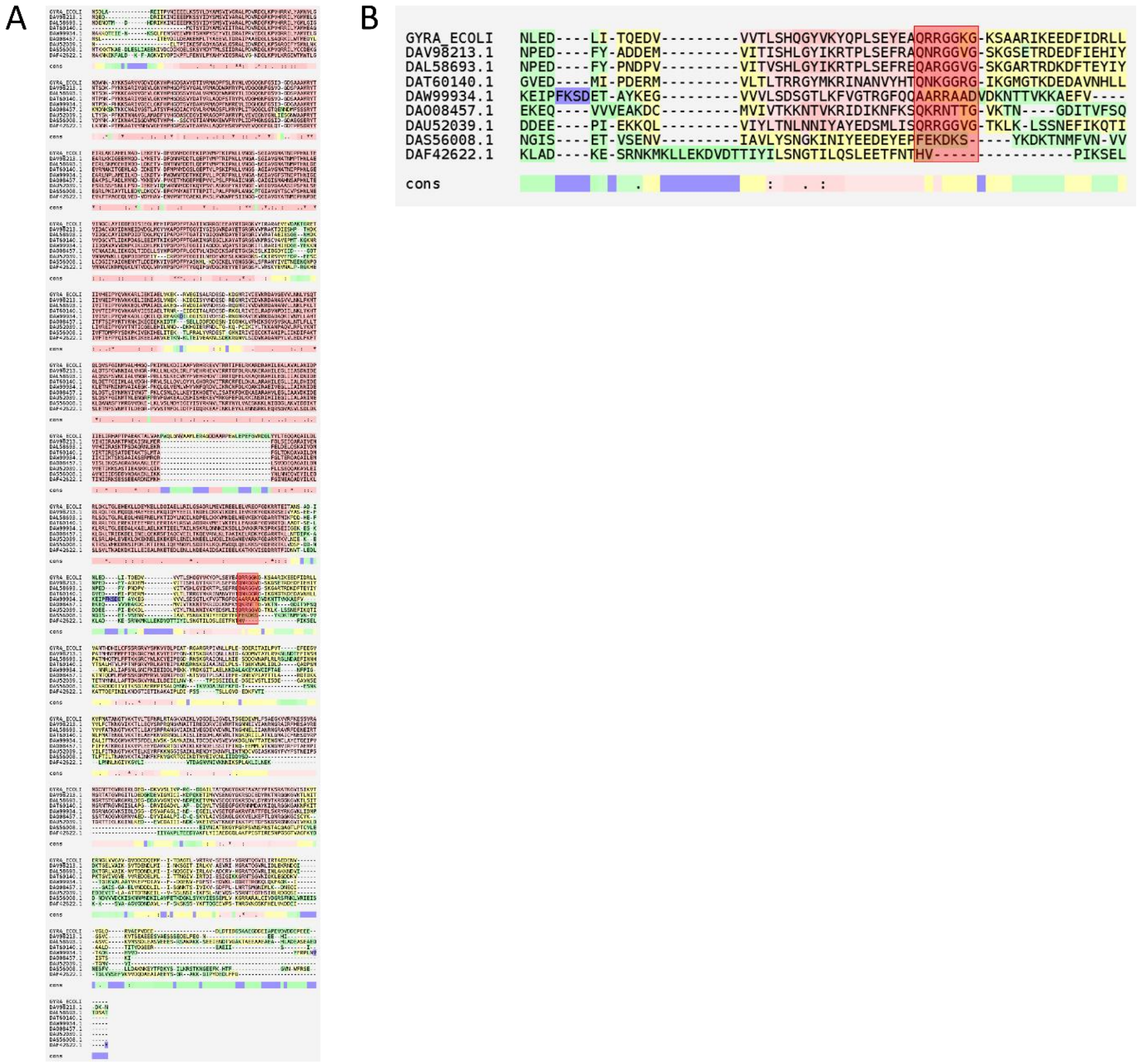

Supplementary figure 8. Alignment of GyrA-like sequences from metagenome assembled viral genomes

BLASTp search was used to identify viral homologs of *E. coli* GyrA (Uniprot ID: P0AES4). Viral sequences showing more than 25 % sequence identity over at least 70 % of sequence length were taken into consideration. This yielded over 100 sequences corresponding to Microviridae, Myoviridae, Siphoviridae and Podoviridae all belonging to Caudales bacteriophages. One or two sequences were selected for each taxonomic group for alignment which was performed using T-coffee web server (Di Tommaso et al. 2011) with default settings.

Part A shows the global alignment with GyrA box (QRRGGKG) highlighted with a red rectangle and part B is enlarged part of the alignment containing the GyrA box. The first sequence is GyrA of *E. coli* which serves as a reference for canonical GyrA protein. Four viral sequences possess the canonical GyrA box motif while in the remaining four sequences the motif is degenerated or missing.

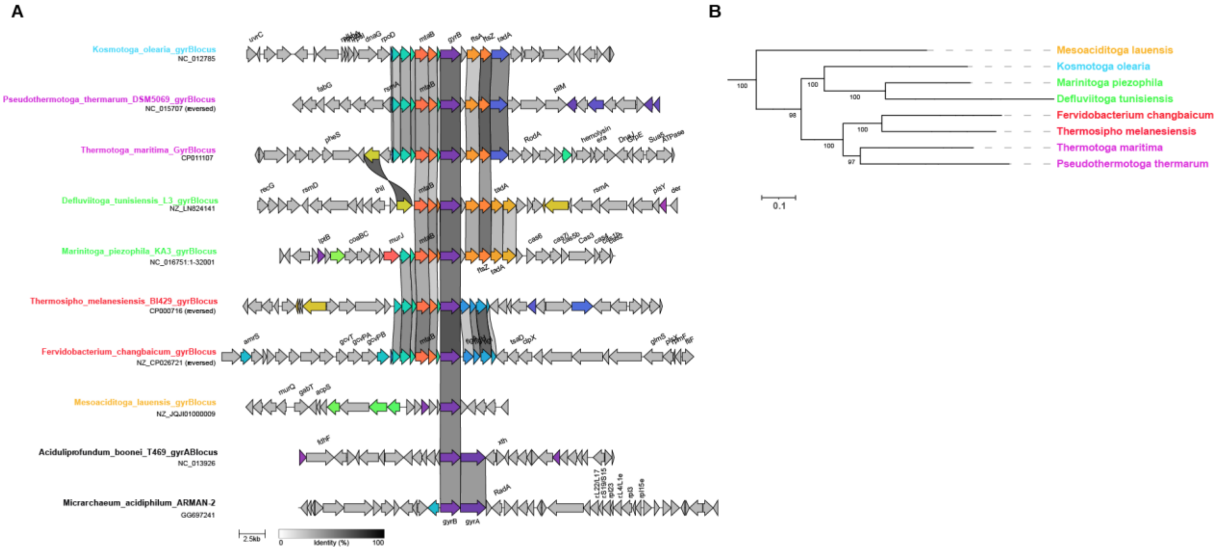

Supplementary figure 9. Synteny analysis of the *gyrB* locus in Thermotogales

A) The genomic context around *gyrB* gene is depicted with each arrow corresponding to a gene. Genes are automatically color coded based on functional annotation. The scale bar at the bottom corresponds to the percentage of identity between proteins encoded by the depicted genes. The drawing is on scale with the scale bar representing 2.5 kbp. The bacterial species names are indicated in color and the same color code is used for the phylogenetic tree shown on the right.

B) Phylogeny of Thermotogales species used in synteny analysis. The phylogenetic tree was automatically generated using PhyloT and the Genome Taxonomy Database and visualized using iTOL (Letunic & Bork 2021). The numbers on the branches correspond to bootstrap values for node support.

A

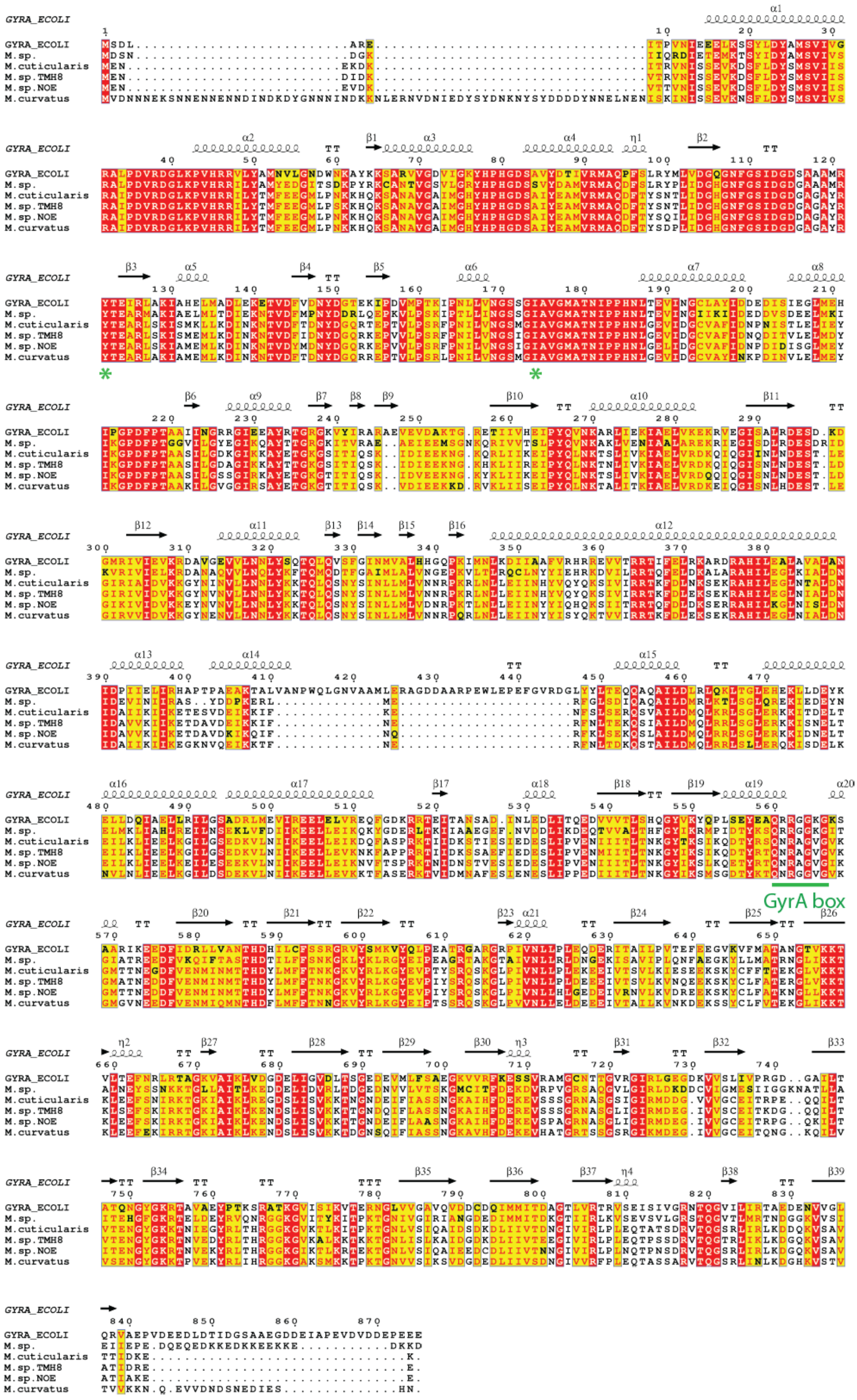
GYRA_ECOLI
M.sp.
M.cuticularis
M.sp.TMH8
M.sp.NOE
M.curvatus
GyrA box

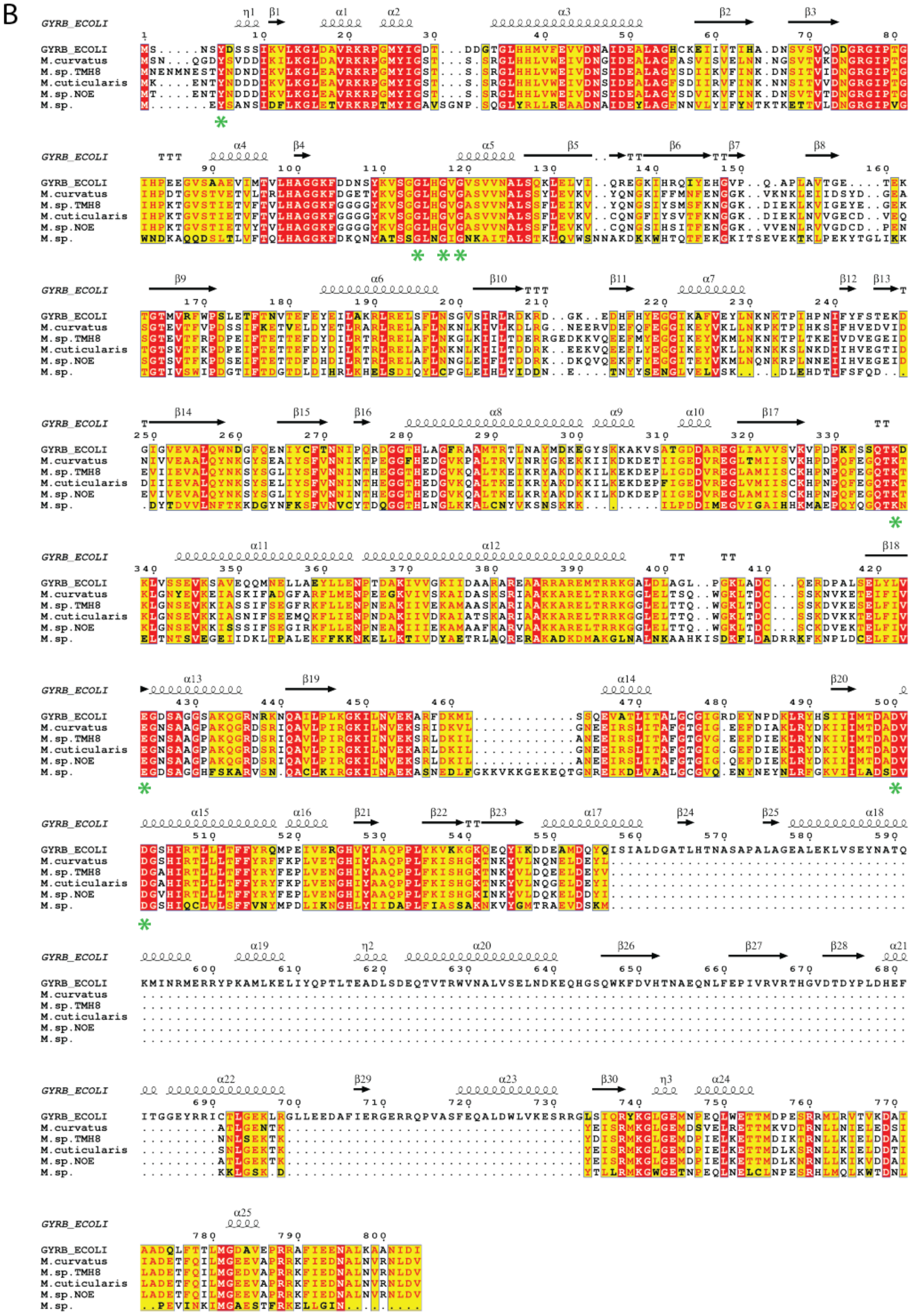

Supplementary figure 10. Sequence alignment of GyrA and GyrB orthologs from *Methanobrevibacter* species.

Sequences were aligned using T-coffee web server (Di Tommaso et al. 2011) and secondary structure information was rendered using ESPript 3.0 (Robert & Gouet 2014). The catalytically important residues and the GyrA box are indicated with green star and green line, respectively. For full description of functionally important residues, see Schoeffler & Berger, 2008.

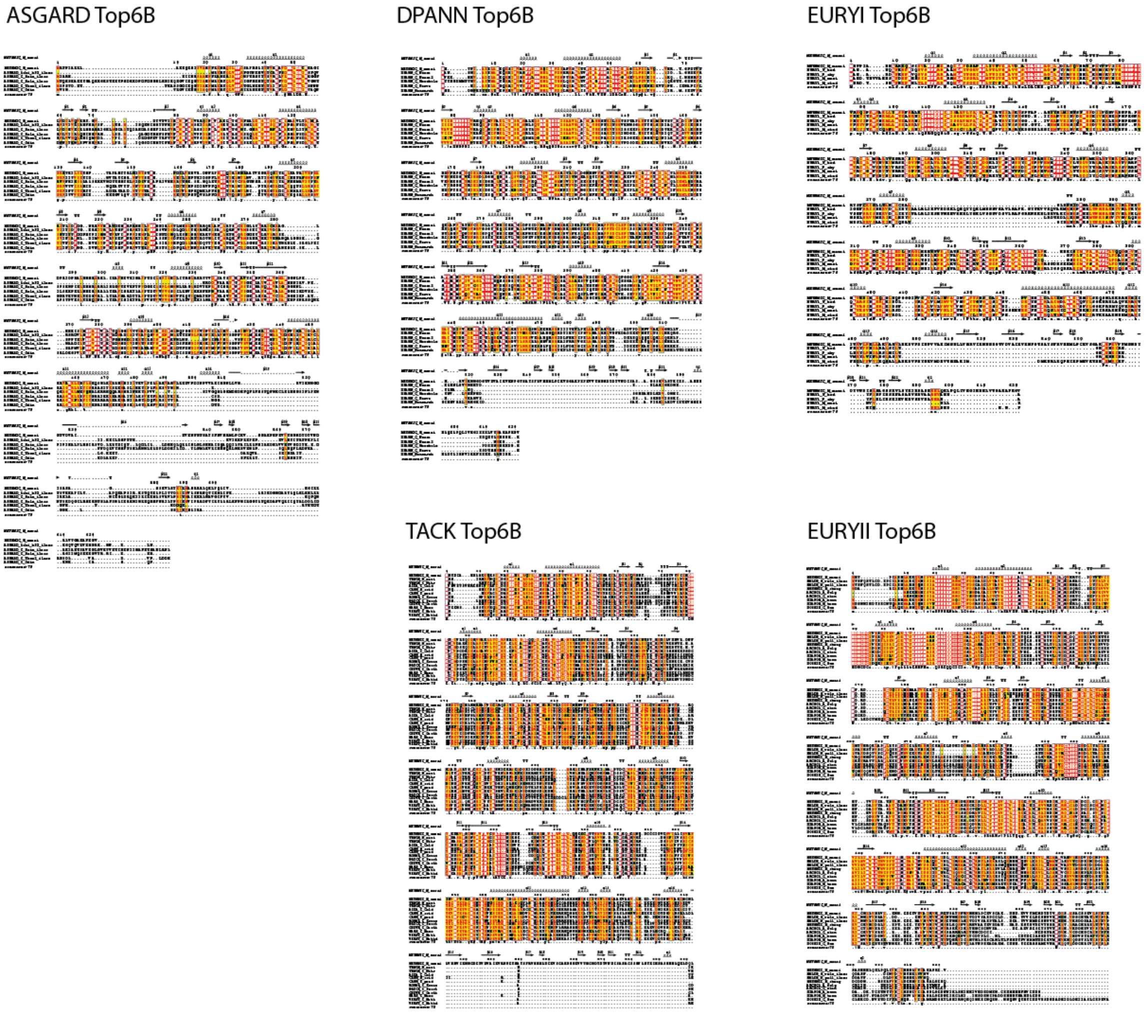

Supplementary figure 11. Alignment of Top6B sequences from major archaeal taxonomic groups.

Sequences from that were used to generate the phylogenetic tree of Top6B orthologs were aligned using T-coffee web server (Di Tommaso et al. 2011). The sequence of *Methanosarcina mazei* (PDB 2Q2E) was used as reference in each alignment in order to render secondary structure information using ESPript 3.0 (Robert & Gouet 2014). Conserved and partially conserved residues are highlighted as red and yellow columns, respectively. Most of the sequence variability between the taxonomic groups is visible at the C-terminal part of Top6B.

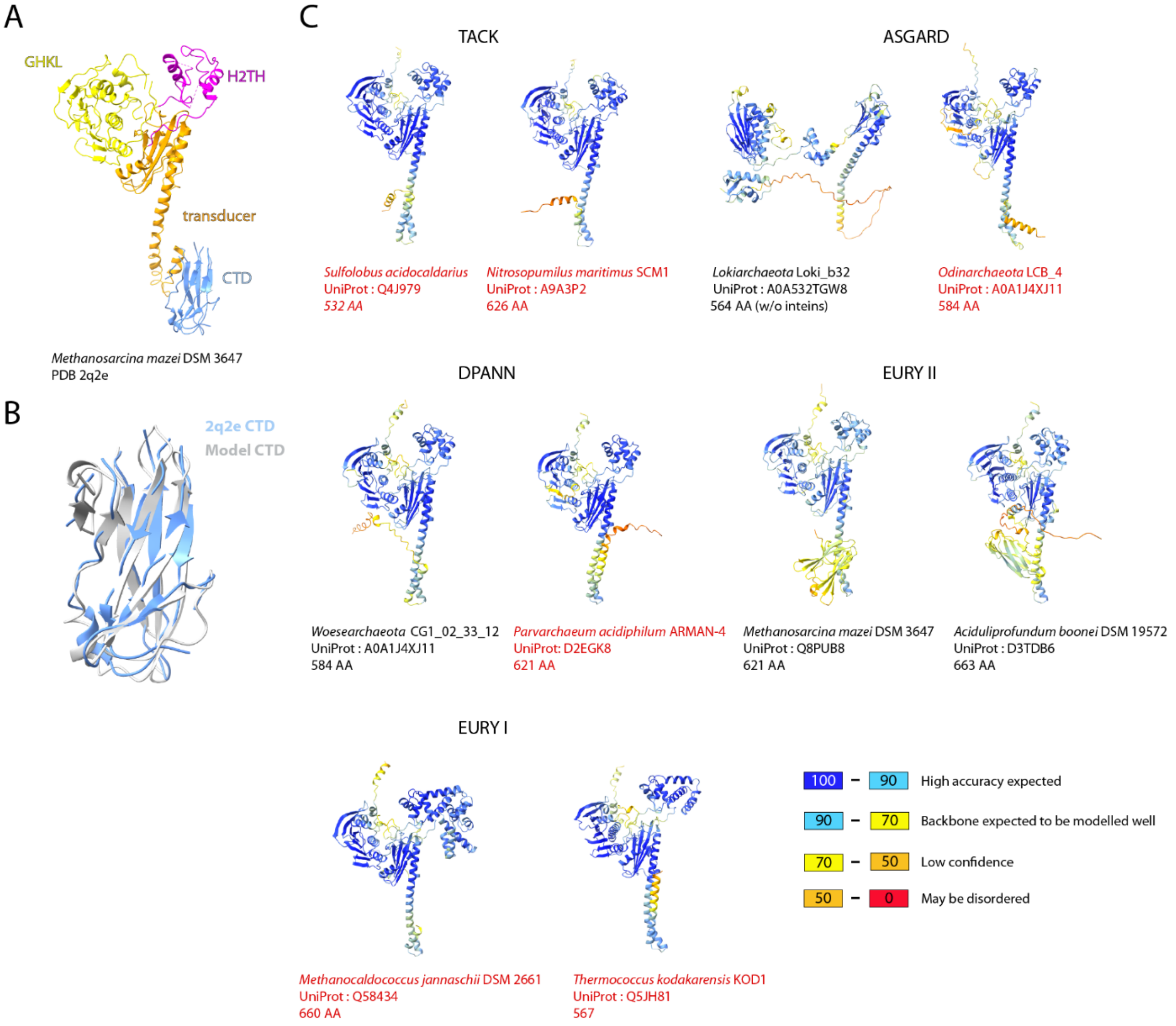

Supplementary figure 12. *De novo* model structures of representative archaeal Top6B orthologs.

A) Domain organisation of Top6B subunit. Key functional modules are indicated with different colours. N-terminal domain is homologous to GHKL-type ATPases, a small helix-2-turn-helix (H2TH) domain is inserted between GHKL and transducer domains.

B) Superposition between the X-ray crystallographic structure of CDT from *M. mazei* (PDB 2q2e) and *de novo* model predicted by AlphaFold2.

C) AlphaFold2-predicted structures of representative Top6B sequences. Red and black lettering indicates absence and presence of gyrase in an organism, respectively. AlphaFold2-predicted structures are coloured by the pLDDT confidence measure.

REFERENCES

Di Tommaso P, Moretti S, Xenarios I, Orobitg M, Montanyola A, Chang JM, Taly JF, Notredame C. 2011. T-Coffee: a web server for the multiple sequence alignment of protein and RNA sequences using structural information and homology extension. *Nucleic Acids Res*. Jul;39(Web Server issue):W13-7. doi: 10.1093/nar/gkr245.

Letunic I, Bork P. 2021. Interactive Tree Of Life (iTOL) v5: an online tool for phylogenetic tree display and annotation. *Nucleic Acids Res*. Jul 2;49(W1):W293-W296. doi: 10.1093/nar/gkab301.

Robert X, Gouet P. 2014. Deciphering key features in protein structures with the new ENDscript server. *Nucleic Acids Res.* Jul;42(Web Server issue):W320-4. doi: 10.1093/nar/gku316.

Schoeffler AJ, Berger JM. 2008. DNA topoisomerases: harnessing and constraining energy to govern chromosome topology. *Q Rev Biophys*. 41(1):41-101.
